# Deciphering Molecular Mechanisms and Diversity of Plant Holobiont Bacteria: Microhabitats, Community Ecology, and Nutrient Acquisition

**DOI:** 10.3390/ijms252413601

**Published:** 2024-12-19

**Authors:** Tomasz Grzyb, Justyna Szulc

**Affiliations:** Department of Environmental Biotechnology, Faculty of Biotechnology and Food Sciences, Lodz University of Technology, Wolczanska 171/173, 90-924 Lodz, Poland; tomasz.grzyb@dokt.p.lodz.pl

**Keywords:** plant holobiont, microbial ecology, bacteria biodiversity, environmental interactions, nutrient acquisition

## Abstract

While gaining increasing attention, plant–microbiome–environment interactions remain insufficiently understood, with many aspects still underexplored. This article explores bacterial biodiversity across plant compartments, including underexplored niches such as seeds and flowers. Furthermore, this study provides a systematic dataset on the taxonomic structure of the anthosphere microbiome, one of the most underexplored plant niches. This review examines ecological processes driving microbial community assembly and interactions, along with the discussion on mechanisms and diversity aspects of processes concerning the acquisition of nitrogen, phosphorus, potassium, and iron—elements essential in both molecular and ecological contexts. These insights are crucial for advancing molecular biology, microbial ecology, environmental studies, biogeochemistry, and applied studies. Moreover, the authors present the compilation of molecular markers for discussed processes, which will find application in (phylo)genetics, various (meta)omic approaches, strain screening, and monitoring. Such a review can be a valuable source of information for specialists in the fields concerned and for applied researchers, contributing to developments in sustainable agriculture, environmental protection, and conservation biology.

## 1. Introduction

Humanity is currently facing the crisis of climate change, the disruption of biogeochemical cycles, the increasing scale of biological invasions, the dramatic loss of biodiversity, and, at the same time, the unprecedented need to feed an ever-growing human population that will reach 9.8 billion people by 2050 [1,2,3,4,5]. There is a need for environmentally friendly solutions, whether in agriculture, conservation biology, or environmental protection. Plant-associated bacteria with plant-growth-promoting (PGP) and plant-growth-inhibiting (PGI) traits can be one of the most desired and promising solutions, offering an opportunity for sustainable agriculture and invasive species management [6,7]. However, a comprehensive understanding of fundamental processes at the environmental and molecular levels regarding plant microbiome is crucial in this case [8,9]. It is often the missing link for numerous applied studies [10]. This phenomenon should be considered not only in the context of the effects of single strains on the phenotype of an individual plant but also in the context of microbial community ecology, plant–plant, plant–microbiome, and intra-microbiome interactions [9,11,12,13].

It is worth noting that the diversity and distribution uncovered by high-throughput molecular techniques over the past decades have profoundly changed our understanding and application of plant–microbe research [9]. Both ecological and reductionist approaches have significantly impacted plant microbiome research, but each is highly limited when used alone [9]. The key is to combine a reductionist approach regarding mechanisms with analyses of community ecology, macroecological patterns, and evolutionary contexts [9]. An example of going beyond the reductionist paradigm is the application of synthetic microbial communities (SynComs) instead of single strains or strain mixtures [9,14,15].

So far, the application of plant-associated bacteria is limited primarily to soil and rhizosphere bacteria. However, the potential use of bacteria from other plant parts—the microbiota of leaves, seeds or flowers, and endophytes, in general—is increasingly being described [16,17,18,19,20]. For example, functional and mechanistic understanding of phyllosphere microbiota at the community level is perceived as a potential key advancement in the next decade [21]. A situation of exceptional under-studying is particularly true for the microbiome of the anthosphere, where no systematic review of the taxonomic structure of the microbiota is available. This most basic level of biodiversity meta-analysis is available for the other compartments but not for the microbiota of flowers.

Therefore, this review aims to update and synthesise information on the ecology of microbial communities, biodiversity, and molecular aspects of bacterial processes related to plant nutrients. The scope of the review includes (i) an explanation of key terminology for omics aspects in plant studies, (ii) a discussion of plants as compartments as microenvironments for the microbiota, (iii) a systematic gathering of data on the taxonomic structure of the floral microbiome, (iv) a discussion of the ecological interactions between microorganisms and microorganisms and plants, (v) a discussion of processes within the plant–microbiome system concerning bioavailability and acquisition of nutrients, nitrogen (N), phosphorus (P), and potassium (K), typical components of synthetic NPK fertilisers and iron (Fe), playing a key role in cell metabolism and interactions in the plant microbiome, (vi) and an establishment of molecular markers specific for nutrient-related processes that can be used in various genetic, metataxonomic, (meta)genomic, and (meta)transcriptomic studies, as well as strain potential screening and biomonitoring. This article provides a solid theoretical foundation for applied research in the era of emerging SynComs.

This review not only updates and synthesises existing knowledge across multiple plant microbiome compartments but also pioneers new insights into underexplored areas, offering a comprehensive foundation for future research. By addressing key ecological and molecular interactions, we aim to inspire innovations in microbial community applications, ultimately driving advances in sustainable agriculture, ecological management, and environmental conservation.

## 2. Terminological Issues in Molecular Diversity Studies

The subject matter of this article encounters several terminological ambiguities that require clarification. Firstly, distinguishing the term “microbiome” from the “microbiota” is the issue. The term “microbiome” was introduced in 1988 by Whipps et al. [22]. Since then, various other definitions have been proposed, including ecological, organism-dependent, genomic/method-driven, or combined definitions [23]. In 2020, Berg et al. revisited the original definition by adding two explanatory sentences, differentiating microbiome from microbiota [23]. The contemporary definition (also adopted in this work) is “The microbiome is defined as a characteristic microbial community occupying a reasonable, well-defined habitat which has distinct physio-chemical properties. The microbiome not only refers to the microorganisms involved but also encompasses their theatre of activity, which results in the formation of specific ecological niches. The microbiome, which forms a dynamic and interactive micro-ecosystem prone to change in time and scale, is integrated into macro-ecosystems including eukaryotic hosts, and here crucial for their functioning and health”. The microbiota, on the other hand, is defined as an assembly of microorganisms—*Bacteria*, *Archaea*, and microbial *Eukaryota*. Abovementioned “theatre of activity” includes “microbial structures, metabolites, mobile genetic elements (e.g., transposons, phages, and viruses), and relic DNA embedded in the environmental conditions of the habitat” [23]. For a more in-depth perspective, see Berg et al. [23].

Another important term is a holobiont. A holobiont is a host (such as a plant) and members of its microbiota, considered a single entity, a unit of selection, subject to co-evolution [24]. A core microbiota is part of a microbiota which is persistent and ubiquitous in almost all the communities associated with a particular host. It contains taxa crucial for host fitness [24]. It is worth noticing that various members of those taxa are used as plant growth promoters: biopesticides; biofertilisers; biostimulants; and biodegradation stimulators [25]. Often, a given species performs several different roles that are difficult to separate. From an eco-evolutionary perspective, all plant–microbiome interactions are considered “means to generate new phenotypes with increased fitness under distinct environmental conditions” [24].

There are two ways in which the terms rhizosphere and phyllosphere are used in scientific articles. In the broader sense, the rhizosphere is the entire belowground part of the plant, and the phyllosphere is the whole aboveground part [26,27,28,29]. In the narrower sense, the rhizosphere is the niche consisting of the soil in the close vicinity of the roots, and the phyllosphere is the leaf niche [11,29]. Therefore, the authors have divided these niches into rhizosphere and phyllosphere sensu lato (s.l.) and sensu stricto (s.s.), as depicted in Figure in Section 3. The terms rhizosphere and phyllosphere without s.s. or s.l. notations are used when it is impossible to determine which meaning was intended by the publication in question.

Terminological confusion also arises when it comes to the use of omics methods for microbiome analysis. The main subject of this confusion is metagenomics. In the past, both marker gene high-throughput sequencing (HTS) and shotgun sequencing of microbial DNA were referred to as metagenomics [30]. Today, it is strongly recommended that those two types of analysis be distinct [30,31]. Metataxonomics is a high-throughput process applied to characterise the microbiota composition and to create metataxonomic trees [31]. To achieve this, HTS of markers such as 16S rDNA, 18S rDNA, and ITS regions is performed [30,31]. On the other hand, the metagenomic approach requires random shotgun sequencing of all DNA extracted from the environmental sample, followed by assembly, mapping, and annotation processes [30,31].

The so-called multi-omic approach may include taxonomics, metataxonomics, genomics, metagenomics, transcriptomics, metatranscriptomics, proteomics, metaproteomics, metabolomics, and metabonomics (the term metabonomics was suggested to avoid the unhandy meta-metabolomics) [31,32]. Multi-omics is synonymous with integrated omics and involves applying multiple omic technologies [33]. The term panomics is occasionally used [34,35]; however, in our opinion, the prefix pan- can be misleading, as it indicates the use of all methods (it is worth comparing this to terms such as pangenome and pangenomics).

Sometimes, the term holo-omics is also used [36,37]. Briefly, holo-omics is a multi-omic approach applied to the study of holobionts. According to the authors, the term has merit, as it is about a specific research objective and a specific subtype of methodology involving the pairing and integration of host and microbiota datasets.

Finally, in this article, scientific names at all taxonomic ranks are set in italics, as postulated by Thines et al. [38]. Names of all bacterial taxa are used according to the List of Prokaryotic names with Standing in Nomenclature (LPSN) [39].

## 3. Plant Compartments as Microenvironments for the Microbiota

**Figure 1:**
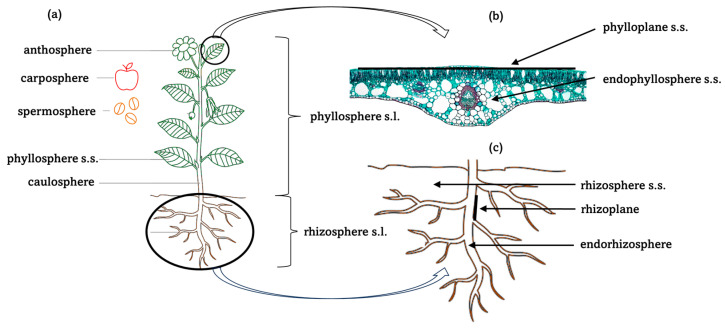
Main types of plant habitats colonised by microorganisms, s.l.—sensu lato; s.s—sensu stricto; (**a**) general microhabitats; (**b**) leaf microhabitats; (**c**) rhizosphere s.l. microhabitats (Graphic elements from u/nnnn (ipuzzle. pl) and Berkshire Community College Bioscience Image Library (CC0, via Flickr) were used).

Plants offer various niches that support the development and abundance of a wide array of microorganisms, such as *Bacteria*, *Archaea*, *Fungi*, and non-fungal microbial *Eukaryota* [24]. There is also a complex mosaic of viruses on, inside, and around the plants [40,41]. The main types of niches and the general rules of nomenclature are presented in Figure 1.

The best-studied plant microhabitat is the rhizosphere s.l. (Figure 1a,c)—the entire underground part of the plant [26,28]. It consists of three main niches: the rhizosphere s.s., that is, the soil directly surrounding the plant’s roots; the rhizoplane—the surface of the roots; and the endorhizosphere—the interior of the roots [11]. In the case of plants in symbiosis with arbuscular mycorrhizal fungi (AMF), beyond the rhizosphere s.s. (or mycorrhizosphere, the rhizosphere s.s. of roots colonised by AMF) extends the hyphosphere—the zone of the surface of AMF hyphae and the soil immediately adjacent to them [42,43,44]. There seems to be a distinct bacterial microbiome of the hyphosphere; however, this is already a zone under the dominant influence of AMF (and not the plant) [42,43,44].

The distribution patterns of the predominant bacterial groups are comparable in both bulk soil and the rhizosphere s.s., as the rhizosphere selects microorganisms from bulk soil to function as a ‘seed bank’, though reducing microbial diversity [11,24,45]. Ling et al. compared 557 pairs of published 16S rDNA amplicon sequences from the bulk soils and rhizosphere in different ecosystems worldwide to generalise bacterial characteristics [45]. Several taxa, *Pseudomonadota*, *Bacteroidota*, and *Actinomycetota*, are typically and consistently enriched in the rhizosphere [45]. This highlights their general adaptation to carbon-rich conditions prevalent in the rhizosphere, promoting high metabolic activity, rapid growth, and propagation. Thus, they are considered to be copiotrophs (r-strategists: opportunistic, fast-growing microbes with high ribosome content, in contrast to oligotrophs (K-strategists)) [45]. On the other hand, *Chloroflexota*, *Acidobacteriota,* and *Nitrospirota* are consistently depleted in the rhizosphere [45]. Moreover, functional predictions indicate a significant enrichment of genes related to organic compound conversion, nitrogen fixation, and denitrification in the rhizosphere. Conversely, there is a notable depletion of genes associated with nitrification [45].

The phyllosphere s.l. (Figure 1a,b) is an open system. It is a complex and dynamic environment, characterised by rapid fluctuations in factors like water, nutrients, and temperature. Additionally, variations in plant species and genotypes contribute to its overall variability [17,21,24,26,28,29]. Phyllosphere s.l. is also associated with high light and UV radiation [46]. Several responses to abiotic stress, such as extracellular polymeric substances (EPS) production, accumulation of osmoprotectants, pigmentation, and activation of antioxidant enzymes, have been reported [17,21].

The microbiota found in the phyllosphere s.l. can originate from various sources, including soil, seeds, and air, occurring at different stages throughout the plant’s growth and development [21,24,29]. Leaf epiphytes primarily originate from within the plant, specifically from seed tissue and the germination environment [17]. Following seedling emergence, microbes are likely introduced by various means, including wind, raindrops, irrigated water, insects, and bioaerosols [17]. Once deposited, their establishment and survival on the leaf surface hinge on crucial microbial traits, such as the ability to acquire foliar nutrients, adhere to the cuticle, and thrive in the leaf microclimate and host leaf characteristics [17].

While the compositions of bacteria associated with roots significantly differ from those found in aboveground communities, both constitute subsets of the microbiota originating from soil communities [21,24]. These subsets are enriched in distinct plant-associated niches, indicating that soil is a shared reservoir for belowground and aboveground plant microbiota [21,24]. For instance, in the case of grapevine, although belowground bacterial communities exhibited notable distinctions from their aboveground counterparts, the communities linked to leaves, flowers, and grapes displayed a higher proportion of shared taxa with soil communities than each other [47].

The bacterial communities in the phyllosphere s.s. of seed plants are predominantly characterised by a few phyla, such as *Pseudomonadota*, *Actinomycetota*, *Bacteroidota*, and *Bacillota* [21,24]. In the case of eudicot plant species (and similarly in monocots), specific bacterial genera dominate, particularly *Sphingomonas* (*Alphaproteobacteria*), *Methylobacterium* (*Alphaproteobacteria*), and *Pseudomonas* (*Gammaproteobacteria*) [21]. The consistent identification of *Sphingomonas* and *Methylobacterium* in leaf phyllosphere microbiotas using the metataxonomics approach seems connected to their adaptation to low nutrient availability on leaf surfaces. The persistence and co-occurrence of various microbial taxa (such as *Sphingomonas*, *Methylobacterium*, *Pseudomonas*, *Pantoea*, and *Variovorax*) within the leaf core microbiome across different plant genotypes, geographical locations, and growing seasons are consistently reported [21].

Microorganisms colonise the endosphere of every plant compartment. The endosphere is the most restricted plant area, with less diverse communities than external parts [24]. Different plant organs are associated with different endophytic bacterial communities in terms of diversity and composition [48].

Bacterial endophytes commonly enter root tissues through active mechanisms and passive processes, such as root cracks or emergence points of lateral roots [27]. The microbiome in the root endosphere is significantly less diverse than in the rhizosphere s.s. and bulk soil [48,49]. This indicates that roots serve as efficient habitat filters, limiting the composition of communities to increasingly specific lineages as environments shift from soil to root systems [48,49]. The two-step rhizosphere s.l. recruitment model has been proposed by Bulgarelii et al. [50]. During the initial differentiation phase, the combination of rhizodeposits and host cell wall characteristics stimulates the growth of organotrophic bacteria, instigating a shift in the soil biome community. In the subsequent step, host genotype-dependent selection, occurring near and within the root corpus, finely adjusts community profiles, fostering microorganisms thriving on the rhizoplane and within plant roots [50].

Bacterial communities within root endophytes are commonly characterised by a dominant presence of *Pseudomonadota* (with *Gammaproteobacteria* standing out as the most diverse and predominant) [51]. *Actinomycetota*, *Bacteroidota*, and *Bacillota* are other phyla frequently encountered as endophytes [48,51]. Additional bacterial phyla, including *Chloroflexota*, *Cyanobacteriota*, *Armatimonadota*, *Verrucomicrobiota*, *Planctomycetota*, and *Nitrospirota*, are commonly observed in the root endosphere, constituting a smaller proportion of the community [48]. *Acidobacteriota* and *Gemmatimonadota* seem absent or rarely occur in the root endosphere despite being significant components of microbial communities in bulk soil [48].

Endophytes in the phyllosphere s.s. are primarily transmitted systemically through the xylem [17]. Alternatively, they may traverse leaf epidermal openings such as stomata, lenticels, and hydathodes. While microbes can utilise any of these pathways for entry, stomata are regarded as the primary gateway to the internal tissues of leaf surfaces [17]. In contrast to the challenges presented by microbial colonisation on the leaf surface, the endosphere compartment poses a distinct set of challenges. Relative to plant surfaces, the endosphere is likely to offer more abundant nutrients and potential protection for microbes against external atmospheric changes, such as UV radiation and moisture fluctuations [21]. However, compared to epiphytes, endophytes are closer to the plant’s immune surveillance mechanisms and defensive compounds. This proximity may limit their potential for multiplication [21].

As there is evidence indicating that bacterial endophytes in plant roots are primarily sourced from the soil, subsequently ascending to stems and leaves through the apoplast in xylem vessels, it is unsurprising that the endomicrobiotas of plant leaves and shoots exhibit significant overlaps with those found in roots, both in terms of taxonomy and functionality [48]. In line with this, *Pseudomonadota*, *Actinomycetota*, and *Bacillota* are the dominant groups in these microbiotas [48].

The aboveground components of phyllosphere s.l. include the phyllosphere s.s., carposphere (fruits), anthosphere (flowers), and caulosphere (stems) (Figure 1a). The anthosphere is unique as this microenvironment has a shorter life span than other plant spheres [52]. Even newly opened flowers are not sterile; they typically harbour microbial presence on various flower tissues [53]. The abundance and composition of microbial communities can vary significantly among flowers. Before anthesis (flower opening), both flower buds and nectar in unopened flowers can contain detectable bacterial and fungal species [53]. Furthermore, the petals of newly opened flowers are found to contain detectable and culturable microbial communities [53].

Microbial incidence and abundance increase over time on individual flowers. Moreover, animal visitors are a significant vector of microorganisms to and among other flowers [53]. For instance, a shared microbiome of bees and flowers has been described [54]. In general, microbes found on flowers can be divided into four groups: those of environmental origin (wind or water dispersed, such as *Micrococcus*); phyllosphere epiphytes (such as *Pseudomonas* and *Sphingomonas*); rhizosphere and phyllosphere endophytes (such as *Erwinia*, *Xanthomonas*, *Streptomyces*); and flower specialists, for whom flowers are a primary habitat. Within bacterial genera, the prominent examples are *Acinetobacter* and *Rosenbergiella* [53]. Today, the anthosphere remains a significantly understudied compartment of the plant microbiome [55,56,57].

It should be noted that the anthosphere lacks a systematic data collection of even a strictly taxonomic structure based on amplicons of markers such as 16S rDNA. One of the aims of this article is to prepare this type of compilation, which is available for other compartments in the literature. The most recent review of the taxonomic structure of the flower microbiota was performed by Aleklett et al. in 2014 [57]. Compilations of the members of flower microbiota have subsequently been published at least twice [52,53], but they analysed other topics. None addressed the composition of the bacteriome determined by culture-independent methods. The authors, therefore, prepared a data collection of the structure of the flower microbiome, presented in Table 1. It includes all studies of the floral microbiome using culture-independent methods from 2015 to September 2024, indexed in the PubMed database and SciSpace AI tool [58]. To the authors’ knowledge, this is the first publication of its kind in the last decade and the first in general in terms of depth of analysis. Publications, published supplementary materials, and, in some cases, published raw data were included in the analysis. Due to particular primer bias in metataxonomic studies, the information regarding amplified 16S rDNA hypervariable regions is also provided.

It is difficult to generalise on a relatively small dataset, on different taxonomic types, with or without percentages. Still, it is certainly noticeable that there is significant variation between species and given structures in flower and cultivation conditions, as well as between-sex phenotypes (in the study by Wei and Ashman [59]).

Like other plant compartments, the spermosphere microbiota can be divided into epiphytes and endophytes (members of the seed endomicrobiota). War et al. collated all available data (as of 2023) on the seed taxonomics diversity [20]. Their study unveiled the connection between seeds of various plant species and bacteria encompassing over 200 genera and 11 phyla. Among these, *Pseudomonadota* emerged as the most prevalent phylum, featuring 125 genera, with *Gammaproteobacteria* being the dominant class, consisting of 55 genera. *Actinomycetota* and *Bacillota* followed with 49 and 36 genera, respectively. *Bacteroidota* and *Acidobacteriota* were less common, represented by 13 and 3 genera [20].

Johnston-Monje et al. have examined seed microbiotas of seventeen academically and economically important plant species [60]. What is essential, they distinguished between seed endomicrobiota and seed epiphytes. Most of the ascribed bacteria were *Pseudomonadota*. Thirty-seven genera were common to the spermoplane, and fifteen were common to the seed endomicrobiota. *Pantoea* and *Enterobacter* were the only genera common to the endomicrobiota of all analysed plant species. They were also common to all spermoplanes, as were *Pseudomonas* and *Bacillus*. Other common bacteria genera were *Massilia* and *Klebsiella* [60].

It seems that only a minor proportion of microbial taxa inhabit the seeds [20]. The limited diversity is ascribed to the dynamic conditions experienced by seeds during their development and maturation. These conditions selectively permit the colonisation of seeds by microbes possessing essential characteristics, including desiccation resistance, endospore formation, cell motility, and enzyme activities such as phytase and amylase activities [20].

In general, microorganisms can be transmitted to seeds either vertically or horizontally. As War et al. [20] summarise that the transmission pathways include (i) the internal pathway, initiated either from the seed or from the soil during germination, culminating in the seed through the passage in the xylem and non-vascular tissues; (ii) the floral pathway through the stigma of the mother plant; and (iii) external pathways where seeds become colonised by microbes from the surrounding environment [20]. The internal pathway is the primary contributor to vertical transmission, while the floral and external pathways play a role in horizontal transmission. It is worth noting that the transfer of microbes through pollen follows the floral route but may sometimes involve vertical transmission [20].

Seed endophytes are particularly intriguing due to their transmission from generation to generation [61]. Their status as seed-borne entities ensures their consistent presence in subsequent plant generations. The endospermosphere is a unique element of the plant holobiont as it relates to the inheritance of the microbiome [62,63,64].

**Table 1:** A compilation of all studies on the total floral microbiome or individual floral structures (excluding pollen) published between 2015 and 2024, indexed in the PubMed database or SciSpace AI tool [58]. The data were based on articles, supplementary materials and, in some cases, published raw data. Percentages have been included where they could be calculated. The aim was to create a summary at the genus and family level; in some cases (in the second part of the table), this was not possible, in which case, the summarised taxa (with the lowest possible taxa level) were marked in the given sections. Order of taxa relative to abundance unless otherwise noted. Names in publications were corrected based on the current guidelines placed in the LPSN [39]. N.A.—not available.

Research Article	Hypervariable Region Sequenced	Plant Host	Sample Type	Taxonomic Criteria 1 and 2	Families	Genera
Aizenberg-Gershtein et al., 2015 * [65]	V4 16S rDNA	*Nicotiana attenuata* Torr. ex S.Watson (WT, nicotine rich)	nectar	(1) families (>1% relative abundance, all OTUs before subsampling) *, (2) genera (>1% relative abundance, after subsampling) *	*Streptococcaceae* (23.82%), *Enterobacteriaceae* (15.91%), *Micrococcaceae* (9.61%), *Staphylococcaceae* (8.71%), *Corynebacteriaceae* (7.44%), *Neisseriaceae* (4.47%), *Actinomycetaceae* (4.33%), *Eubacteriales_Incertae_Sedis_XI* (3.08%), *Fusobacteriaceae* (2.41%), *Pasteurellaceae* (1.58%), *Aeromonadaceae* (1.54%), *Caryophanales_Incertae_Sedis_XI* (1.49%), *Moraxellaceae* (1.38%), *Paracoccaceae* (1.16%), *Veillonellaceae* (1.16%)	*Lactococcus* (24.51%), *Yersinia* (14.36%), *Brevibacterium* (7.73%), *Nesterenkonia* (7.69%), *Kingella* (4.61%), *Cetobacterium* (2.87%), *Mannheimia* (1.42%), *Anaerococcus* (1.38%), *Acidithiobacillus* (1.11%), *Kocuria* (1.06%)
		*Nicotiana attenuata* Torr. ex S.Watson (nicotine-biosynthesis silent)			*Streptococcaceae* (19.57%), *Staphylococcaceae* (9.49%), *Neisseriaceae* (6.66%), *Corynebacteriaceae* (6.48%), *Eubacteriales_Incertae_Sedis_XI* (5.52%), *Micrococcaceae* (5.3%), *Enterobacteriaceae* (3.91%), *Actinomycetaceae* (3.58%), *Fusobacteriaceae* (2.52%), *Pasteurellaceae* (2.33%), *Flavobacteriaceae* (1.91%), *Pseudomonadaceae* (1.64%), *Veillonellaceae* (1.57%), *Bifidobacteriaceae* (1.55%), *Prevotellaceae* (1.52%)	*Lactococcus* (20.68%), *Kingella* (8.02%), *Brevibacterium* (7.02%), *Nesterenkonia* (4.18%), *Yersinia* (3.28%), *Cetobacterium* (3.24%), *Anaerococcus* (1.87%), *Mannheimia* (1.85%), *Bifidobacterium* (1.52%), *TM7_genus_incertae_sedis* (1.45%), *Paraprevotella* (1.36%), *Kocuria* (1.29%), *Cellvibrio* (1.15%), *Parvimonas* (1.06%)
		*Nicotiana glauca* Graham (WT, anabasine rich)			*Enterobacteriaceae* (54.84%), *Streptococcaceae* (8.47%), *Eubacteriales_Incertae_Sedis_XI* (5.67%), *Corynebacteriaceae* (5.44%), *Staphylococcaceae* (3.02%), *Micrococcaceae* (2.23%), *Moraxellaceae* (2.16%), *Pasteurellaceae* (1.76%), *Neisseriaceae* (1.62%), *Pseudomonadaceae* (1.55%), *Caryophanales_Incertae_Sedis_XI* (1.29%), *Fusobacteriaceae* (1.18%), *Actinomycetaceae* (1.16%)	*Yersinia* (57.25%), *Lactococcus* (8.21%), *Brevibacterium* (5.19%), *Anaerococcus* (2.94%), *Nesterenkonia* (1.35%), *Mannheimia* (1.34%), *Kocuria* (1.25%), *Cellvibrio* (1.18%), *Kingella* (1.12%), *Alkanindiges* (1.05%)
Junker and Keller, 2015 [66]	V4 16S rDNA	*Metrosideros polymorpha* Gaudich.	nectar	classified indicator genera used for a successful classification of samples to plant organs	N.A.	*Actinomyces*, *Dietzia*, *Rothia*, *Actinomycetospora*, *Hymenobacter*, *Chryseobacterium*, *Bacillus*, *Geobacillus*, *Streptococcus*
			stamina			*Mycobacterium*, *Lactobacillus*, *Mycoplana*, *Limnobacter*, *Limnohabitans*, *Bdellovibrio*, *Buchnera*, *Acinetobacter*, *Alkanindiges*, *Lysobacter*
			styles			*Cloacibacterium*, *Pedobacter*, *Agrobacterium*, *Magnetospirillum*, *Erwinia*, Candidatus *Portiera*, *Marinomonas*, *Pseudomonas*, *Lysobacter*
Allard et al., 2016 [67]	V1-V3 16S rDNA	*Solanum lycopersicum* L.	flowers in general (plants in 5 different rows)	(1) families (>1% relative abundance in at least 1 sample type)	*Lysobacteraceae* (24.7–66.1%), *Enterobacteriaceae* (2–22.8%), *Pseudomonadaceae* (3.3–18.2%), *Sphingomonadaceae* (2.5–12.3%), *Rhizobiaceae* (0.6–11.8%), *Shewanellaceae* (2.1–8.2%), *Comamonadaceae* (0.1–3.4%), *Methylobacteriaceae* (0.1–2%), *Phyllobacteriaceae* (0–1.5%), *Nitrobacteraceae* (0–1.1%)	N.A.
Allard et al., 2018 [68]	V1-V3 16S rDNA	*Solanum lycopersicum* L.	flowers in general (netted and non-netted)	(1) families (>1% relative abundance, mean value), (2) genera (>1% relative abundance, mean value)	*Lysobacteraceae* (34.8%), *Sphingomonadaceae* (14.8%), *Shewanellaceae* (14.0%), *Pseudomonadaceae* (7.3%), *Microbacteriaceae* (7.2%), Unclassified *Pseudomonadales* (6.7%), *Rhizobiaceae* (5.9%), *Enterobacteriaceae* (3.7%), *Comamonadaceae* (2.4%)	Unclassified *Lysobacteraceae* 1 (31.6%), *Shewanella* (14.0%), *Sphingomonas* (13.6%), *Microbacterium* (7.0%), *Pseudomonas* (7.0%), Unclassified *Pseudomonadales* 1 (6.7%), *Agrobacterium* (5.9%), Unclassified *Enterobacteriaceae* 1 (3.6%), *Luteimonas* (2.6%), Unclassified *Comamonadaceae* 1 (2.3%)
Purahong et al., 2018 [69]	V3-V4 16S rDNA	*Actinidia chinensis* Planch., *Actinidia deliciosa* (A.Chev.) C.F.Liang and A.R.Ferguson	flowers in general (treated and untreated with *Pseudomonas syringae* pv. *actinidiae*)	(1) families (>1% relative abundance in at least 1 sample), (2) genera (>1% relative abundance in at least 1 sample)	*Acetobacteraceae* (40.92–82.04%), *Acidimicrobiaceae* (1.10–11.05%), *Alcaligenaceae* (1.29–5.56%), *Anaerolineaceae* (0.16–4.75%), *Bacillaceae* (0.97–4.39%), *Alicyclobacillaceae* (0.23–4.30%), *Aerococcaceae* (1.02–3.51%), *Chitinophagaceae* (0.68–3.27%), *Enterobacteriaceae* (0.87–2.96%), *Actinomycetaceae* (0.33–2.09%), *Bacteroidaceae* (0.51–2.02%), *Campylobacteraceae* (0.12–1.55%), *Armatimonadaceae* (0.06–1.25%), *Coriobacteriaceae* (0.20–1.16%), *Burkholderiaceae* (0.22–1.07%), *Bifidobacteriaceae* (0.09–1.07%)	*Roseomonas* (10.22–72.68%), Unclassified *Acetobacteraceae* 1 (0.81–14.30%), *Ilumatobacter* (1.10–11.05%), Unclassified *Acetobacteraceae* 2 (1.31–9.21%), *Acidisoma* (0.39–7.42%), Unclassified *Acetobacteraceae* 4 (2.28–4.49%), *Alicyclobacillus* (0.23–4.30%), Unclassified *Anaerolineaceae* 1 (0.00–4.30%), Unclassified *Acetobacteraceae* 5 (0.03–4.08%), *Advenella* (0,00–3.63%), *Aerococcus* (0.03–3.43%), Unclassified *Acetobacteraceae* 3 (0.38–3.34%), Unclassified *Acetobacteraceae* 6 (0.09–3.22%), *Bacillus* (0.96–3.05%), *Facklamia* (0.03–2.16%), *Bacteroides* (0.51–2.02%), *Campylobacter* (0.12–1.55%), Unclassified *Bacillaceae* 1 (0.00–1.41%), Unclassified *Alcaligenaceae* 1 (0.12–1.28%), *Candidimonas* (0.16–1.10%), *Mobiluncus* (0.09–1.05%)
Kim et al., 2019 [70]	V1-V3 16S rDNA	*Fragaria × ananassa* Duchesne	flowers in high-diversity, low *Botrytis cinerea* infection period, weeks 0–12	(1) families (>1% relative abundance in at least 1 week)	*Moraxellaceae* (0–93.12%), *Bartonellaceae* (0.14–61.92%), *Lysobacteraceae* (0.01–43.35%), *Pseudomonadaceae* (0.01–30.01%), *Burkholderiaceae* (0–27.26%), *Corynebacteriaceae* (0–16.82%), *Nitrobacteraceae* (0.06–6.79%), *Oxalobacteraceae* (0–6.79%), *Streptomycetaceae* (0.06–3.56%), *Staphylococcaceae* (0–2.28%), *Propionibacteriaceae* (0–1.48%), *Streptococcaceae* (0–1.39%), *Sphingomonadaceae* (0–1.14%)	N.A.
			flowers in low-diversity, higher *Botrytis cinerea* infection incidence period, weeks 14–24		*Pseudomonadaceae* (97.49–99.96%)	N.A.
Ryniewicz et al., 2019 [71]	V3-V4 16S rDNA	*Polemonium caeruleum* L.	nectar	(1) dominant families, (2) dominant genera	*Moraxellaceae*, *Phyllobacteriaceae*, *Sphingomonadaceae*, *Methylobacteriaceae*, *Arcobacteraceae*	*Acinetobacter*, *Phyllobacterium*, *Sphingomonas*, *Methylobacterium*, *Arcobacter*
Warren et al., 2020 [72]	V4 16S rDNA	*Asclepias curassavica* L.	nectar	(2) dominant genera	N.A.	*Acinetobacter*, *Neokomagataea*
Gaube et al., 2021 [56]	V4 16S rDNA	*Ranunculus acris* L.	flowers in general	(1) families (>1% relative abundance), (2) genera (>1% relative abundance)	*Pseudomonadaceae* (25.30%), *Enterobacteriaceae* (19.70%), *Sphingobacteriaceae* (9.50%), *Moraxellaceae* (8.21%), *Cytophagaceae* (6.54%), *Lactobacillaceae* (3.84%), Unclassified *Mycoplasmatales* (3.09%), *Mycoplasmataceae* (2.94%), *Oxalobacteraceae* (2.47%), *Streptococcaceae* (1.40%), *Comamonadaceae* (1.22%), *Methylobacteriaceae* (1.04%)	*Pseudomonas* (25.30%), Unclassified *Enterobacteriaceae* 2 (17.67%), *Sphingomonas* (6.45%), *Hymenobacter* (5.90%), *Acinetobacter* (5.24%), *Lactobacillus* (3.84%), Unclassified *Mycoplasmatales* 1 (3.09%), Unclassified *Moraxellaceae* 1 (2.97%), *Mesoplasma* (2.94%), Unclassified *Sphingomonadaceae* 1 (2.00%), *Arsenophonus* (1.82%), *Duganella* (1.47%), *Lactococcus* (1.40%), Unclassified *Gammaproteobacteria* 1 (1.32%), Unclassified *Comamonadaceae* 2 (1.22%), *Methylobacterium* (1.04%)
		*Trifolium pratense* L.			*Pseudomonadaceae* (35.75%), *Enterobacteriaceae* (26.55%), *Sphingomonadaceae* (9.14%), *Oxalobacteraceae* (4.30%), *Methylobacteriaceae* (2.68%), *Flavobacteriaceae* (2.47%), *Lysobacteraceae* (2.25%), *Rhizobiaceae* (2.08%), *Sphingobacteriaceae* (1.81%), *Lactobacillaceae* (1.76%), *Comamonadaceae* (1.4%), *Cytophagaceae* (1.34%), *Acetobacteraceae* (1.26%)	*Pseudomonas* (35.75%), Unclassified *Enterobacteriaceae* 2 (26.51%), *Sphingomonas* (8.18%), *Duganella* (3.65%), *Methylobacterium* (2.68%), *Chryseobacterium* (2.43%), *Lactobacillus* (1.76%), *Stenotrophomonas* (1.70%), *Pedobacter* (1.66%), Unclassified *Comamonadaceae* 2 (1.40%), Unclassified *Acetobacteraceae* 7 (1.26%), Unclassified *Rhizobiaceae* 1 (1.06%), *Rhizobium* (1.02%)
Hayes et al., 2021 [55]	V5-V6 16S rDNA	*Helianthus tuberosus* L.	petal epiphytes	(2) genera (at least 1 ASV with >1% relative abundance)	N.A.	alphabetically: *Aeromicrobium*, *Brevundimonas*, *Buttiauxella*, *Chryseobacterium*, *Curtobacterium*, *Erwinia*, *Frigoribacterium*, *Methylobacterium*, *Oerskovia*, *Pantoea*, *Pseudomonas*, *Rahnella*, *Rhizobium*, *Rhodococcus*, *Sanguibacter*, *Stenotrophomonas*, *Verticiella*
		*Verbesina alternifolia* (L.) Britton ex Kearney			N.A.	alphabetically: *Aeromicrobium*, *Buttiauxella*, *Curtobacterium*, *Frigoribacterium*, *Oerskovia*, *Pantoea*, *Pseudomonas*, *Rhizobium*, *Sanguibacter*, *Stenotrophomonas*, *Verticiella*
Ruraż, Przemieniecki, Błaszak, et al., 2023 [73]	V3-V4 16S rDNA	*Phelipanche arenaria* (Borkh.) Pomel	stigmas (mature and immature)	(2) genera (given percentage in at least 1 sample type)	N.A.	eudominant (35.01%+): *Pantoea*, dominant (10.01–35.0%): *Pseudomonas*, *Luteibacter*, subdominant (5.01–10.0%): *Sphingomonas*, *Rhodococcus*, *Serratia*, *Stenotrophomonas*, rare (2.01–5%): *Pseudarthrobacter*, *Methylorubrum*, *Duganella*, *Pedobacter*, occasional (1.01–2%): *Variovorax*, *Aeromicrobium*, *Aureimonas*, *Xylophilus*, *Blastococcus*, *Nocardioides*, *Enhydrobacter*, *Lactobacillus*, *Skermanella*, *Corynebacterium*, *Paenibacillus*
Schaeffer et al., 2023 [74]	V4-V5 16S rDNA	*Prunus amygdalus* Batsch	anthers	(1) families (>1% relative abundance)	*Pseudomonadaceae*, *Enterobacteriaceae*, *Moraxellaceae*, *Microbacteriaceae*, *Halomonadaceae*	N.A.
			nectar		*Pseudomonadaceae*, *Enterobacteriaceae*, *Oxalobacteraceae*, *Microbacteriaceae*	
			petals		*Pseudomonadaceae*, *Enterobacteriaceae*, *Lactobacillaceae*, *Moraxellaceae*, *Bacillaceae*, *Microbacteriaceae*, *Methylobacteriaceae*, *Oxalobacteraceae*, *Comamomonadaceae*, *Acetobacteraceae*, *Neisseriaceae*, *Orbaceae*	
Boutin et al., 2024 [75]	V5-V6 16S rDNA	*Malus x domestica* (Suckow) Borh.	flowers, in general	(2) genera (>1% relative abundance)	N.A.	*Bacillus* (16%), *Hymenobacter* (15%), *Deinococcus* (7%), *Pseudomonas* (6%), *Erwinia* (6%), *Sphingomonas* (5%), *Massilia* (4%), *Amnibacterium* (4%), *Frondihabitans* (4%), *Spirosoma* (4%), *Curtobacterium* (3%), *Pantoea* (2%), *Methylobacterium* (2%)
Tiusanen et al., 2024 [76]	V4 16S rDNA	*Apiaceae* (2 species), *Asteraceae* (12 species), *Balsaminaceae* (1 species), *Campanulaceae* (1 species), *Ericaceae* (4 species), *Fabaceae* (5 species), *Geraniaceae* (1 species), *Hypericaceae* (1 species), *Lamiaceae* (3 species), *Onagraceae* (1 species), *Orobanchaceae* (3 species), *Plantaginaceae* (2 species), *Polygonaceae* (1 species), *Primulaceae* (2 species), *Ranunculaceae* (1 species), *Rosaceae* (2 species), *Rubiaceae* (2 species), *Solanaceae* (1 species), *Violaceae* (2 species), studied altogether	flowers, in general	(1) families (>1% relative abundance), (2) genera (>1% relative abundance)	*Erwiniaceae* (55.3%), *Enterobacteriaceae* (51.8%), *Pseudomonadaceae* (46.5%), *Sphingomonadaceae* (34.2%), *Oxalobacteraceae* (21.1%), *Methylobacteriaceae* (12.3%), *Moraxellaceae* (11.4%), *Comamonadaceae* (7.0%), *Aurantimonadaceae* (6.1%), *Mycoplasmataceae* (6.1%), *Hafniaceae* (6.1%), *Microbacteriaceae* (5.3%), *Orbaceae* (5.3%), *Hymenobacteraceae* (4.4%), *Morganellaceae* (4.4%), *Rickettsiaceae* (4.4%), *Spiroplasmataceae* (4.4%), *Ehrlichiaceae* (3.5%), *Rhizobiaceae* (3.5%), *Sphingobacteriaceae* (3.5%), *Yersiniaceae* (3.5%), *Paenibacillaceae* (2.6%), *Dysgonomonadaceae* (1.8%), *Halomonadaceae* (1.8%), *Lactobacillaceae* (1.8%), *Rhodanobacteraceae* (1.8%), *Weeksellaceae* (1.8%), *Lysobacteraceae* (1.8%)	N.A.
					**Orders**	**Genera**
Sauer et al., 2021 [77]	V5-V6 16S rDNA	*Achillea millefolium* L.	bud epiphytes	(1) classes (>1% relative abundance), (2) main genera (approx. 5% relative and more)	*Pseudomonadales*, *Burkholderiales*, *Sphingomonadales*, *Enterobacterales*, *Hyphomicrobiales*, *Caryophanales*, *Actinomycetales*, *Cytophagales*	*Pseudomonas*, *Sphingomonas*, *Janthinobacterium*, *Duganella*
			bud endophytes		*Burkholderiales*, *Pseudomonadales*, *Sphingomonadales*, *Hyphomicrobiales*, *Enterobacterales*, *Actinomycetales*, *Caryophanales*, *Cytophagales*	*Pseudomonas*, *Janthinobacterium*, *Sphingomonas*, *Duganella*, *Herbaspirillum*
			mature flower epiphytes		*Pseudomonadales*, *Enterobacterales*, *Lysobacterales*, *Sphingomonadales*, *Paenibacillales*, *Rickettsiales*, *Actinomycetales*, *Burkholderiales*, *Mycobacteriales*, *Lactobacillales*, *Rhodospirillales*, *Eubacteriales*	*Pseudomonas*, *Serratia*, *Pantoea*, *Sphingomonas*
			mature flower endophytes		*Enterobacterales*, *Pseudomonadales*, *Sphingomonadales*, *Rhodospirillales*, *Hyphomicrobiales*, *Actinomycetales*, *Caryophanales*, *Lysobacterales*, *Mycobacteriales*, *Lactobacillales*	*Nissabacter*, *Pectobacterium*, *Pseudomonas*
					**Phyla**	**Genera**
Zarraonaindia et al., 2015 ** [47]	V4 16S rDNA	*Vitis* (L.)	flowers, in general	(1) phyla (>1% relative abundance) **, (2) dominant genera	*Pseudomonadota* (98%), *Bacillota* (>1%) **	*Pseudomonas* (61.8%), *Erwinia* (25.2%s)
Jiménez Elvira et al., 2022 [78]	V4 16S rDNA	*Alpinia japonica* (Thunb.) Miq.	flower epiphytes	(1) dominant phyla, (2) dominant genera	*Pseudomonadota* (avg. 47.8% of prokaryotic sequences), *Actinomycetota*	*Pseudomonas*, *Erwinia*
Ruraż, Przemieniecki, and Piwowarczyk, 2023 [79]	V3-V4 16S rDNA	*Orobanche alsatica* Kirschleger, *Orobanche bartlingii* Griseb.	stigmas (mature and immature)	(1) main phyla, (2) main genera (given percentage in at least 1 sample type)	*Actinomycetota*, *Pseudomonadota*, *Bacillota*, *Bacteroidota*	eudominant (40.01%+): *Cellulosimicrobium*, dominant (10.01–40.0%): *Pantoea*, *Pseudomonas*, *Sphingomonas*, subdominant (5.01–10.0%): *Paenibacillus*, rare (2.01–5%): *Sphingomonas*, *Serratia*
Xi et al., 2023 [80]	V4 16S rDNA	*Citrus sinensis* L. Osbeck × *Poncirus trifoliata* L.	flowers, in general	(1) main phyla, (2) genera (>1% relative abundance)	*Pseudomonadota*, *Bacillota*, *Cyanobacteriota*, *Bacteroidota*	*Acinetobacter*, *Sphingomonas*, *Gilliamella*, *Aquabacterium*, *Snodgrassella*, *Pseudomonas*, *Bacillus*, *Massilia*, *Streptomyces*, *Actinoplanes*
					**Phyla**	**Families**
Steven et al., 2018 [81]	V4 16S rDNA	*Malus x domestica* (Suckow) Borh.	stigmas	(1) phyla (>1% relative abundance), (2) dominant families	*Pseudomonadota*, *Cyanobacteriota*, *Bacteroidota*	*Pseudomonadaceae*, *Enterobacteriaceae*
			stamens			
			receptacles			
			petals		*Pseudomonadota*, *Cyanobacteriota*, *Bacteroidota*, *Chloroflexota*	
Wei and Ashman, 2018 [59]	V4 16S rDNA	*Fragaria chiloensis* (L.) Mill, *Fragaria virginiana* Duchesne ssp. *platypetala* (Rydb.) Staudt, *Fragaria × ananassa* Duchesne subsp. *cuneifolia* (Nutt. ex Howell) Staudt	flowers divided by sexual phenotype	(1) dominant phyla	*Pseudomonadota*, *Bacteroidota*, *Actinomycetota*	N.A.
Cui et al., 2021 [82]	V4 16S rDNA	*Malus x domestica* (Suckow) Borh.	stigmas (of flowers treated and untreated with Erwinia amylovora at various time points)	(1) main phyla (mean values), (2) dominant families (mean values)	*Pseudomonadota* (94.3%), *Cyanobacteriota* (3.6%)	*Enterobacteriaceae* (70.0%), *Pseudomonadaceae* (26.2%)
					**Classes**	**Orders**
Rebolleda Gómez and Ashman, 2019 [83]	V4 16S rDNA	*Erythranthe guttata* (Fisch. DC.) G.L.Nesom	stamens (pollination exclusion treatments and control)	main orders (in at least 1 sample type)	N.A.	*Pseudomonadales*, *Bacteroidales*, *Eubacteriales*, *Actinomycetales*, *Enterobacterales*, *Caryophanales*, *Lactobacillales*, *Hyphomicrobiales*, *Burkholderiales*, *Cytophagales*, *Sphingomonadales*, *Rhodospirillales*, *Neisseriales*
			petals (pollination exclusion treatments and control)			
			styles (pollination exclusion treatments and control)			
Kim et al., 2023 [84]	V3-V4 16S rDNA	*Brassica juncea* (L.) Czern., *Veronica polita* Fr., *Capsella bursa-pastoris* (L.) Medik., *Lamium amplexicaule* L., *Lamium purpureum* L., *Taraxacum platycarpum* Dahlst, *Potentilla indica* (Andrews) Th.Wolf, *Forsythia koreana* (Rehder) Nakai, *Vicia villosa* Roth, *Chelidonium majus* L. subsp. *asiaticum* (H. Hara) Ohwi, *Prunus jamasakura* (Makino) Siebold ex Koidz., *Viola mandshurica* Wilhelm Becker	flowers, in general	(1) most dominant bacterial orders (mean values)	N.A.	*Burkholderiales* (18.13% ± 11.07%), *Enterobacterales* (17.50% ± 20.80%), *Sphingomonadales* (15.24% ± 11.00%), *Caulobacterales* (8.98% ± 6.87%), *Caryophanales* (8.69% ± 20.44%)
Lee et al., 2024 [85]	V3-V4 16S rDNA	*Malus x domestica* (Suckow) Borh.	flowers in different developmental stages: bud, pop, full	(1) main classes	alphabetically: *Acidimicrobiia*, *Actinomycetes*, *Alphaproteobacteria*, *Bacilli*, *Bacteroidia*, *Betaproteobacteria*, *Cyanpohyceae*, *Gammaproteobacteria*, *Phycisphaerae*, *Terriglobia Thermoleophilia*	N.A.
					**Phyla**	**Classes**
Massoni et al., 2021 [86]	V5-V7 16S rDNA	*Arabidopsis thaliana* (L.) Heynh., Col-0	flowers, in general (grown in various conditions and with two types of soil inocula)	(1) dominant phyla, (2) dominant classes	*Pseudomonadota*, *Actinomycetota*	*Gammaproteobacteria*, *Alphaproteobacteria*, *Actinomycetes*

* data between families and genera differ (as families were counted based on all OTUs and genera—after sampling); ** non-*Pseudomonadota* abundance highly variable in time (15% in early time points, decreasing up to <1%).

## 4. Plant Holobiont from the Microbial Community Ecology Perspective

### 4.1. On Ecological Processes Shaping Plant Microbiome Diversity and Composition

To fully understand the ecological processes leading to microbiota assembly, Vellend’s synthetic theory must be considered [87,88,89,90]. His conceptual synthesis answered a “mess” of multiple theories in community ecology. This synthesis assumes that both deterministic and stochastic processes are key factors in shaping ecological communities [87]. Deterministic processes involve nonrandom, niche-based, organism-trait-dependent mechanisms, including abiotic filtering and various biological interactions [91]. Stochastic processes in ecology are random changes in community structure in relation to species identities and functional traits [91].

Vellend stated that parallel to four evolutionary processes governing population genetic diversity, i.e., selection, genetic drift, mutation, and gene flow, there are four classes of mechanisms shaping ecological communities: selection; ecological drift; speciation; and dispersal [87]. The selection represents deterministic fitness differences, ecological drift—stochastic changes in abundance; speciation—creating new species; and dispersal—the spatial movement of organisms [87]. Nemergut et al. suggested replacing speciation with diversification, as evolutionary change can alter community dynamics even without creating new species [92]. Diversification is generally a stochastic process but can possess several deterministic components [91]. Dispersal cannot be definitively classified as deterministic or stochastic [91]. This theory can also be applied to microbial ecology [91,93] and plant microbiome studies [88,89], as described below.

**Figure 2:**
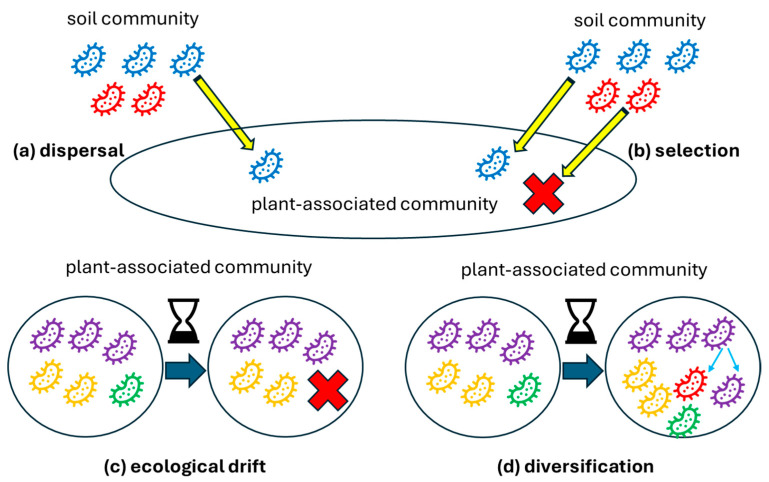
Schematic representation of the four eco-evolutionary processes shaping community assembly, using soil and plant-associated communities as examples, inspired by schemes by Cordovez et al. [88]; (**a**) dispersal, a spatial process with both deterministic and stochastic properties; (**b**) selection, representing deterministic changes in the community composition; (**c**) ecological drift, representing stochastic changes in taxa abundance; (**d**) diversification, representing evolutionary change (see text for more information).

Dispersal (Figure 2a) is based on the movement of the microorganism between different communities. However, it is not only the composition of the microbiota being moved that matters but also the timing of its arrival. The effects of this chronology are referred to as priority effects. Priority effects can be based on facilitation or inhibition, e.g., niche preemption or modification [88]. In addition to horizontal transmission, microbiome inheritance (i.e., vertical transmission) may possibly ensure that specific microbes are passed from one plant generation to the next, providing a consistent microbial community that can be established in the early plant’s life.

Environmental selection (Figure 2b) is conceptually defined as the consequence of biotic and abiotic influences that lead to fitness differences among individuals or species [87,88]. This definition also includes, for example, selection by plant genotype or developmental stage [88]. It is crucial to recognise that a portion of microbial taxa in plant microbiomes may not be assembled directly due to selection. This is because selection requires time to shape microbial abundances toward a stable state [88]. One has to remember, though, that high dispersal rates can lead to the homogenisation of distinct local communities in the soil and plant microbiomes, thereby diminishing the impact of selection [88]. Moreover, although selection filters out less viable taxa, microorganisms capable of dormancy state can evade it. Additionally, vertical transmission may potentially provide a “pre-adapted” set of microbes, possibly giving specific taxa a selective advantage that influences how they interact with local environmental filters.

Ecological drift (Figure 2c) is responsible for random population fluctuations through stochastic birth and death events, occurring independently of species identity [87,88]. For instance, in some plant compartments (such as the endosphere or phyllosphere), overall population sizes are lower than, e.g., in the rhizosphere [9,88]. As less abundant taxa are particularly vulnerable to extinction, the taxonomic structure may change in overall small populations due to the ecological drift.

Diversification (Figure 2d) is here understood as the process of generating new genetic variations in a given population [9,88]. Cordovez et al. point out that it is the most neglected process in structuring plant microbiome. This is due to our limited understanding of how scales influence microbial diversity, as well as our limited understanding of how to investigate this [88]. It seems that the structure of the microbiome is influenced by diversification not only of plant-associated microbes but also by diversification in the bulk soil, as this is the seed bank for the plant microbiota. Furthermore, in general, the process of horizontal gene transfer (HGT) is an additional variable [88]. Microbial inheritance may also influence diversification, as vertically transmitted microbes may potentially undergo evolutionary changes within the host over multiple generations, thus co-evolving with the plant.

Metacommunity theory is valuable for understanding microbial ecology as well [89]. This theory integrates local- and broad (regional)-scale processes that influence community assembly. It assumes that a given community structure (e.g., microbiomes of different plant compartments) is a result of specific processes that occur within the local community, i.e., biotic interactions and abiotic constraints, and the process of dispersal that links different communities [88,94,95]. What is essential is that metacommunity theory explicitly acknowledges that static snapshots of plant microbiomes are not solely the outcomes of local-scale processes at a given time [88]. Instead, the structure of the microbiome emerges from the dynamic interaction of multiple-scale processes in the system, collectively contributing to community historical contingency. This involves considering the impact of the order and timing of past events on community assemblies [88].

### 4.2. On Bacteria-Mediated Plant Processes

Plant–microbe associations were pivotal in the initial colonisation of land by the ancestors of terrestrial plants, and they continue to play a crucial role in plant well-being [96]. In other words, plant evolution is believed to be driven by interactions with microorganisms [97,98]. Although the permanent associations are only part of the interactions within the plant holobiont, at least the part of the holobiont composed of the plant and the core microbiota is seen as the unit of the selection and adaptation processes [11,97].

The plant microbiota can be regarded as a facilitating element that contributes additional genes to the host, participating in the adaptation to specific local environmental conditions [11]. Here, the authors suggest distinguishing six essential areas of microbial facilitation that are (i) nutrient acquisition, (ii) phytohormone level modulation, (iii) abiotic stress tolerance, (iv) biocontrol and plant defence system modulation, (v) microbial homeostasis, and (vi) facilitation of plant–microbe interactions. At the same time, it is important to stress that a complete separation of these areas is impossible as they intermingle, and even a single metabolite can affect several of them.

Regarding nutrient acquisition, in the case of bacteria, it is about fixation, solubilisation, oxidation, reduction, and sequestration of essential nutrients, notably increasing the availability of nitrogen, phosphorus, iron, and potassium [6,99,100,101,102]. In the case of phytohormones, it is a matter of direct or indirect modulation, especially auxins, cytokinins, gibberellins, and ethylene levels [24,51,103,104,105].

Common types of abiotic stress mitigated by the microbiome are, for instance, drought stress, salt stress, flooding, anoxia, and cold stress [6,106]. The impact on the plant safety is at least twofold. Firstly, microorganisms can combat pathogenic microorganisms in various—direct and indirect—ways. Secondly, they can trigger plant responses such as induced systemic resistance response [24,105].

The fifth area deals with microbial homeostasis, and the sixth area deals with the facilitation of plant–microbe interactions. In plants, it is still difficult to define a ’healthy microbiota’ and to draw a line between dysbiosis and eubiosis [107]. Briefly, dysbiosis occurs when there is abnormal microbiome abundance and reduced microbial diversity, and it has a negative impact on plant health; eubiosis is a state of microbiota homeostasis with the maintenance of typical host processes [21]. The terms ’hub’ and ‘keystone’ microorganisms should be introduced here. Hub microorganisms are microbes exhibiting significantly higher connectivity in co-occurrence network analyses than other groups, as determined by centrality measurements like degree, betweenness, and closeness centrality [24]. In other words, hub microorganisms can impact community structure through strong biotic interactions with the host or other microbial species rather than solely relying on their elevated abundance [24]. Some hub species may also be ‘keystone species’—microorganisms capable of exerting potent direct and indirect influences on microbiome assembly, serving as intermediaries between the plant and its associated microbiome [24]. Such organisms are presumably responsible for maintaining the microbiome in eubiosis and its transition to meliorbiosis (a state of homeostasis with altered microbiota associated with a positive impact on plant health under stressful conditions) [21].

It should be emphasised that although the mechanisms of fitness enhancement have been best described for the rhizosphere s.l., they are not limited to the microbiome of this compartment. Bacteria commonly described as plant-growth-promoting are also found in the phyllosphere s.s., the spermosphere, and various endospheres [16,17,20,21,51,61,63,108,109,110].

Naturally, a positive impact is not the only possible balance of bacteria–plant interactions. The microbiome appears to consist of a continuous spectrum of plant-impacting traits, including mostly commensals but occasionally pathogens or mutualists [21,97]. One significant distinction between commensal and pathogenic microbes lies in the pathogen’s capacity to overcome the population restriction mechanisms imposed by the plant host, resulting in uncontrolled proliferation and detrimental effects on the host’s well-being. This capability is attributed to pathogenicity/virulence genes, typically absent in commensal members of the phyllosphere microbiota [21]. In an evolutionary context, it is commonly theorised that pathogens evolved from their plant-associated commensal ancestors by acquiring a comprehensive set of traits related to pathogenesis [21]. On the contrary, since the evolution from commensals to pathogens involves gaining numerous traits, it is also reasonable to anticipate that specific commensals might possess some, though not all, of the genes associated with pathogenicity/virulence. Genome analyses of microbiota indeed support this observation [21].

Rather than categorising microorganisms into distinct lifestyles as commensals, pathogens, or mutualists, it is crucial to recognise that these classifications exist on a continuum [97]. As Mesny et al. point out, the concept of pathogenicity should be viewed as a continuous variable, termed pathogenic potential, encompassing factors such as host damage, time, and virulence [97]. Microorganisms with low pathogenic potential, often referred to as opportunistic pathogens, may (or may not) infrequently cause disease, while those with high pathogenic potential are considered virulent, obligate pathogens [97]. Moreover, a certain level of “virulence” is necessary for the colonisation of plant tissues, with disease establishment being restrained by plant immunity and environmental factors [21,97].

The classic disease triangle foresees that disease develops from the interplay of three main factors: host genetics (susceptibility); pathogen genetics (virulence); and environmental conditions [97,111]. As the microbiome directly and indirectly influences the development of pathogens, it should be perceived as a fourth dimension [111]. The disease development is influenced by the degree of microbial colonisation, which depends on the holobiont’s composition, shaped by environmental stimuli [97]. Importantly, pathogens are integral components of holobionts regardless of their infection strategy [97].

Disruptions in immunity or permissive environmental conditions can lead to disease development, depending on the pathogenic potential of microbiota members. Thus, defence responses targeting all plant-colonizing microorganisms, including those with performance-promoting effects, are essential for plant survival and fitness [21,97,111]. The classical view that immunity evolved to fight particular pathogens is imprecise at least; immunity should be understood as an organism’s ability to withstand any microbial invader that may cause damage, encompassing all members of their microbiota [9,97].

### 4.3. On Bacteria–Microbe Interactions Within Plant Holobiont

Depending on the analysis, it is assumed that plants colonised the land somewhere between the middle Cambrian and middle Ordovician (approximately between 515 and 473 Ma) [112,113]. The first plant–fungal associations are older than 400 Ma [114]. In the case of nitrogen-fixing bacteria, it is over 100 Ma, and symbioses with *Cyanobacteriota* could be even older [114,115]. Yet, this is only a modest fraction of the existence of *Bacteria*, *Archaea*, and microbial *Eukaryota* and the interactions between them, as life on Earth originated at least 3.5 Ga, and eukaryogenesis took place at least 1.65 Ga [12,116,117]. Such microbe–microbe interactions also take place within the plant microbiome.

Antagonistic interactions include resource competition, contact-dependent competition, predation/parasitism and contact-independent competition [12,24]. An example of resource competition is the siderophore production, discussed in detail in Section 6. Contact-dependent competition is based on the excretion of toxic effectors directly into the target cells by using diverse secretion systems, such as type VI secretion system (T6SS), used in plant microenvironments both against *Bacteria* and *Eukaryota*, by various *Alphaproteobacteria*, *Betaproteobacteria* and *Gammaproteobacteria*, or type III secretion system (T3SS), used against *Fungi* and *Oomycota*, e.g., by *Mycetohabitans rhizoxinica* [12,118,119].

In the case of microorganisms, predation is often hardly distinguishable from parasitism [120]. One example is bacterial mycophagy, targeted against various fungi, such as saprotrophic ones in the rhizosphere s.l. [12,121]. Bacteria, such as *Bdellovibrio* spp., present in the rhizosphere s.l., can also prey on other bacteria [12]. Finally, bacteria themselves fall prey to microbial *Eukaryota*, such as *Cercozoa* [122].

Contact-independent competition is based on synthesising various antimicrobial compounds; bacteria target other bacteria and microbial *Eukaryota*, such as fungi. The compounds mentioned are predominantly antibiotics and enzymes, including those targeting a wide range of fungal phytopathogens [12]. Moreover, some produced bacterial volatile organic compounds (VOCs) inhibit the growth of plant-associated fungi and oomycetes [12].

In plant microenvironments, not only antagonistic but also cooperative microbe–microbe interactions take place. These can be divided into nutritional interdependencies, biofilm formation, molecular signalling, enhanced dispersal, and endosymbiosis [12]. In the case of nutritional interdependencies, non-useful nutrients and secreted metabolites may serve as carbon or nitrogen sources for neighbouring microbes, fostering a balanced bacterial population conducive to further growth [118]. A prime example of this cooperative metabolic strategy is observed in the soybean rhizosphere, where the *Bacillus cereus* strain produces peptidoglycan that stimulates the growth of species belonging to *Cytophagales* and *Flavobacteriales* [12,118].

Biofilms are probably the most common way the microbes live in association with plants [118]. Those can be single-species bacterial biofilms or multispecies, including multidomain ones—mixed bacterial and fungal biofilms [118,123]. Those consortia are based on the division of labour, providing a selective advantage for the microorganisms involved, such as protection from contact-dependent and contact-independent competition, activation of high-density-dependent enzymatic processes, and HGT [12,124].

Microbial signalling is primarily associated with the quorum sensing (QS) phenomenon, i.e., the regulation of gene expression due to differences in cell-population density [125]. The most known signalling molecules—autoinducers—are acyl–homoserine lactones (AHLs) in the case of Gram-negative bacteria and autoinducer oligopeptides and AIP (autoinducer peptide), CSF (competence or sporulation factor), or CSP (competence-stimulating peptide) in the case of Gram-positive bacteria [118]. Besides the QS mechanism, different compounds, such as VOCs, serve as signalling molecules [118].

The best-known examples of enhanced dispersal are so-called “fungal highways”. The mechanism of these highways is based on the fact that bacteria use hyphae of filamentous *Eukaryota* as a vehicle to disperse efficiently, especially when there is a water deficit [12]. However, the relationship between bacteria and fungi can go even deeper when endosymbiosis occurs. Microscopic fungi have their own microbiome; these bacteria are mostly taken from the environment but are also subject to vertical transmission via fungal spores [12]. This phenomenon has been described for several plant-associated fungi (e.g., *Rhizophagus*, *Rhizopus*, *Gigaspora*, *Ustilago*, *Laccaria*, *Mortierella*); endosymbiotic bacteria belong primarily to the *Burkholderiaceae* and *Bacillaceae* [12]. Fungal endosymbionts increase the fitness of the fungal host, sometimes providing an essential element to facilitate the colonisation of plants by fungi and even spore production [12,126].

Generally, it is crucial to remember that microbe–microbe interactions are vital for plant growth promotion and disease suppression or facilitation.

## 5. Molecular Diversity of NPK Acquisition

### 5.1. Nitrogen

Earth’s atmosphere contains approximately 78% nitrogen [127]. Yet, it is primarily present as dinitrogen gas (N_2_), an incongruous source for plants and most macro- and microorganisms [127,128]. Plants depend on converted forms of nitrogen, such as nitrate (NO_3_^−^) or ammonium (NH_4_^+^), which can be absorbed from the soil [128]. Nitrogen is the most common limiting nutrient for plant growth, and it is one of the main factors driving the use of synthetic fertilisers [129].

Biological nitrogen fixation (BNF) is a phenomenon found only in some members of the *Bacteria* and *Archaea* domains [128]. Nitrogenase enzyme complex is required for this process. It consists of two component metalloproteins, the iron (Fe) protein (the dinitrogenase reductase, component II) and—in the majority of cases—the molybdenum−iron (MoFe) protein (the dinitrogenase, component I) [127,130,131]. Instead of the MoFe protein, the complex can also be formed by homologous vanadium–iron (VFe) or iron-only (FeFe) proteins [130,132]. The reaction stoichiometry of nitrogen fixation is presented in Table 2. The reaction carried out by nitrogenase is highly energy-demanding [132,133]. Moreover, both components of the nitrogenase complex are extremely sensitive to oxygen and can be rapidly and irreversibly inactivated when the oxygen concentration is too high [102].

The direct product of nitrogen fixation is ammonia (NH_3_). As NH_3_ in high concentrations is toxic to cells; in free-living diazotrophic bacteria, it is assimilated to glutamate by the glutamine synthetase/glutamate synthase pathway [135]. In the case of symbiotic and associative bacteria (see below), NH_3_ is excreted and rapidly assimilated into amino acids by plant enzymes [135]. NH_3_ can also enter the soil solution, where it is converted and/or assimilated by other microorganisms.

Nitrogenase enzyme complex is encoded either by *nif* (MoFe nitrogenase), *vnf* (VFe nitrogenase), or *anf* (FeFe nitrogenase) gene clusters. Every known diazotroph possesses at least one nitrogenase isoform, with some (e.g., *Azotobacter vinelandii* and *Rhodopseudomonas palustris*) even possessing all three [134,136]. The MoFe dinitrogenase is α_2_β_2_-type heterotetramer, in which subunit α is encoded by *nif*K and subunit β by *nif*D, whereas the dinitrogenase reductase (Fe protein) is a γ_2_-type homodimer encoded by *nif*H [132,136]. In the case of alternative isoforms, VFe and FeFe dinitrogenases are α_2_β_2_δ_2_-type hexamers, encoded by *vnf*HDGK *anf*HDGK genes, and subunit δ (encoded by *vnf*G/*anf*G) is essential for nitrogenase complex to function [136].

At least nine genes appear to be required for the synthesis of a functional nitrogenase complex in bacteria—the conserved in *Paenibacillus polymyxa* WLY78 cluster *nif*BHDKENX*hes*A*nif*V transduced into *Escherichia coli* cells enabled nitrogen fixation with *nif*B or T7 promoter [137]. In contrast, the insertion of only six core genes (*nif*BHDKEN) was not sufficient to synthesise a functional nitrogenase; seven genes (*nif*BHDKEN*nif*V) enabled only low nitrogenase activity, and eight genes (with deletion of either *nif*X or *hes*A) led to nitrogen fixation with a 50% reduced efficiency [137].

The best-known transcriptional regulation system of the nif gene cluster is the NifA-NifL system from A. vinelandii, where NifA is an activator and NifL—anti-activator. This mechanism is sensitive to nitrogen limitation—*Pseudomonadota* sense intracellular nitrogen status as the ratio between 2-oxoglutarate (2-OG) and glutamate [134]. The homologs of NifA can be identified in almost all-known diazotrophic *Pseudomonadota*; NiFL is less frequent [136]. Yet, the regulation mechanisms vary among different microorganisms, even when both NifA and NifL are present (to read more about the NifA-NifL, see [134,136]).

Nitrogen fixation by diazotrophs is the primary nitrogen source in terrestrial and aquatic biospheres [138,139]. It is estimated that natural abiotic processes (lightning) are responsible for 5 Tg N year^−1^, terrestrial natural BNF 52–130 Tg N year^−1^, marine and aquatic BNF 120 Tg N year^−1^, agricultural BNF 60 Tg N year^−1^, and industrial sources are 120 Tg N year^−1^ for the Haber–Bosch process and fossil fuel combustion 40 Tg N year^−1^ [139,140,141]. Of an average annual nitrogen fixation of 397–475 Tg N, as much as 220 Tg N is human-made; nevertheless, the BNF is responsible for 232–310 Tg N, including 172–250 Tg N from non-anthropogenic processes. All this highlights the fundamental importance of biological nitrogen fixation.

Nitrogenase stands as an example of an evolutionary singularity, as life evolved a single mechanism, a single enzyme (in three isoforms) capable of facilitating a critical metabolic process [138]. In the following, the diversity of diazotrophic organisms in modern ecosystems concerning the plant microbiome will be presented.

**Figure 3:**
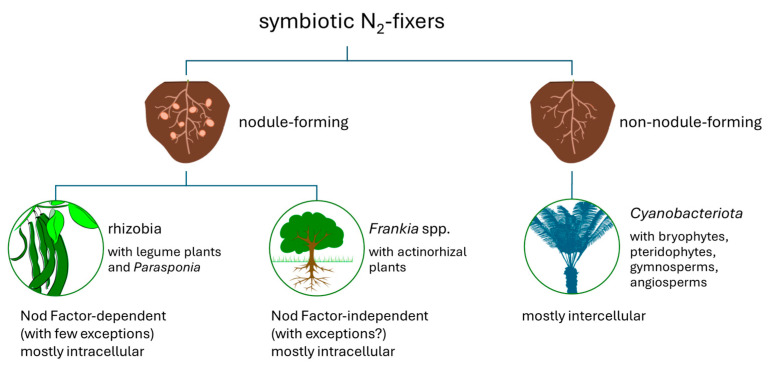
Three groups of symbiotic N2-fixers (graphic elements from PNGWing (non-commercial DMCA), Pixabay (CC0), PhyloPic (CC0) and Nefronus (CC BY-SA 4.0, via Wikimedia Commons) were used).

Three major different groups of nitrogen-fixing bacteria are distinguished: symbiotic N_2_-fixers (SNF; understood as endophytic mutualists); associative N_2_-fixers (ANF); and free-living N_2_-fixers (FLNF) [128]. Symbiotic fixers (Figure 3) typically have the oxygen defence organisation provided by the plant counterpart [128,142]. The two major groups of this interaction are rhizobia associated with legume plants or non-legume *Parasponia* plants and *Frankia* spp. in actinorhizal symbiotic relationships with approx. 25 different genera of woody plants [102,128,142,143].

All known rhizobia are *Pseudomonadota*. There are at least 16 genera in seven families of *Alphaproteobacteria* (α-rhizobia) [144]. Moreover, there are at least four genera in two families in *Betaproteobacteria* (β-rhizobia) and potentially some in *Gammaproteobacteria* (γ-rhizobia) [144]. Details of the so-far-described rhizobia are presented in Table 3.

The third case of symbiotic N_2_-fixers constitutes the symbioses of plants with *Cyanobacteriota*. Symbiotic cyanobacteria are primarily found in the genus *Nostoc*, though a few other *Nostocales* genera, such as *Anabaena*, *Trichormus*, *Calothrix*, and *Chlorogloeopsis*, also contain symbiotic species [145]. In general, some members of *Cyanobacteriota* utilise energy from photosynthesis to enable nitrogen fixation. As photosynthesis involves oxygen emission, protecting nitrogenase from oxygen is particularly challenging in cyanobacteria. Different strategies are adopted: spatial separation of N_2_ fixation and photosynthesis (using specialised cells, such as heterocysts and diazocytes), temporal separation of N_2_ fixation and photosynthesis, combined separation, or even loss of photosynthetic capability [146]. It is worth noticing that although symbiotic *Cyanobacteriota* species are capable of photosynthesis, they are predominantly photosynthetically inactive in a symbiotic state, relying on the host as a carbon source donor [147].

Most cyanobacterial symbioses are intercellular, but *Nostoc* sp. forms an intracellular symbiosis with mosses of the genus *Sphagnum* and angiosperms of the genus *Gunnera* [128,145,147]. Intercellular mutualistic interactions are observed for cyanobacteria interacting with bryophytes (liverworts and hornworts), pteridophytes (notably with the genus *Azolla*), and gymnosperms (*Cycadaceae*) [145,147].

Rhizobia–legume, rhizobia–*Parasponia* symbioses, and *Frankia* symbioses are associated with nodule formation. Rhizobia nodules are known to arise on roots, stems, and aquatic plants [142]. The key molecular driver of this symbiotic relationship is the Nod Factor (NF), a diffusible lipochitooligosaccharide molecule synthesised by rhizobia and recognised by LysM receptor kinases [148]. Briefly, when legume plants require nitrogen, they emit flavonoids, such as luteolin, apigenin, or daidzein [142]. Flavonoids compatible with a specific species of rhizobia bind to a transcription factor NodD [142,145,148]. This factor regulates the genes responsible for producing the NF, a lipochitooligosaccharide (LCO), that is secreted [148]. Some *nod* genes, notably, a *nod*ABCD gene cluster, are found within all rhizobia [149,150]. The *nod*ABC genes are responsible for producing the lipooligosaccharide core common to all NFs and are essential for nodulation [149,150]. Genes related to host specificities, like *nod*E, *nod*L or *nod*M, *nod*P, and *nod*X, determine the specific modifications to NFs [149,150]. Alterations in these genes can lead to shifts in the range of hosts that rhizobia can infect. NFs are identified by receptors that possess extracellular LysM domains [148]. Moreover, as noted by Via et al., there seems to be a two-stage mechanism that involves the recognition of both NF and exopolysaccharides (EPS sugars) to maintain the infection within the root hair [148].

Around 75% of legume plants use root hair entry, and around 25% use the intercellular entry mode via crack entry, which can be either constitutive or conditional [151]. The molecular aspects of intercellular infection remain poorly known. It seems that NF perception and signalling are essential for intercellular infection. Yet, NF-independent rhizobial nodule formation has been described in the genus *Aeschynomene* [143,151].

Far less is known about the nodulation mechanisms of *Frankia* spp. *Frankia,* which generally differ from rhizobia as they can also fix nitrogen outside of the roots, and rhizobia cannot (however, there are exceptions, such as *Azorhizobium caulinodans*) [128,143]. *Frankia* can act both intracellularly and intercellularly [133,143,152]. The signalling and genetic machinery behind the formation of *Frankia* nodulation is still unknown. Flavonoids play a crucial role in actinorhizal symbiosis [145]. Additionally, auxin may function as a signalling molecule, participating in the regulation of communication between *Frankia* and plants [145]. Most *Frankia* do not possess *nod* gene clusters, though it has been described for a few *Frankia* cluster II members [143,152,153]. Purified rhizobial NFs do not themselves trigger a response in actinorhizal plants, which points out significant distinctions between rhizobial- and *Frankia*-interacting mechanisms [143,152]. Different factors have been reported in clusters I and III Frankia, notably RHDF (root–hair-deformation factor) and NINA (*NIN* activating factor). RHDF is a relatively small hydrophilic molecule resistant to heat and chitinase treatment [152]. NINA factors can induce the expression of the symbiotic gene *NIN* [152,154]. The *NIN* gene encodes a transcription factor that plays a pivotal role in nodulation [152,154]. Notably, it is expressed during pre-infection stages in both legumes and actinorhizal plants, triggered by bacterial signalling molecules [152]. While rhizobial NFs are amphiphilic chitin-based molecules, NINA, akin to RHDF, is hydrophilic and resistant to chitinase [152]. NINA is similar to RHDF but appears to have a distinct size [152,154].

In some cases, plants interact with diazotrophic bacteria found in the rhizosphere, on the surface of plant tissues, and with endophytes through casual associations but without lasting interdependence [155]. Such interactions are called associative nitrogen fixation and have been modelled mostly for grasses [128,156]. ANF is considered agronomically significant, e.g., in the case of sugarcane, sweet potato, paddy rice, and maize; it is responsible for approx. 60% of yearly sugarcane’s N supply [128,157]. However, especially in the rhizosphere, ANF and free-living bacteria are hard to distinguish [155]. The ANF bacteria presumably release fixed N after the lysis of bacterial cells; while most, e.g., crop plants, cannot form SNF interactions, they can benefit from the ANF and FLNF [157,158,159]. Most root-associative diazotrophs belong to *Alphaproteobacteria* (e.g., *Azospirillum*, *Gluconacetobacter*), *Betaproteobacteria* (e.g., *Azoarcus*, *Burkholderia*, *Derxia*, *Herbaspirillum*), and *Gammaproteobacteria* (e.g., *Azotobacter*, *Klebsiella*, *Pantoea*, *Pseudomonas*, *Serratia*) [160]. However, members of other classes, such as *Bacilli* (*Paenibacillus*), *Actinomycetes* (*Frankia*), and *Cyanophyceae* (*Nostoc*), were also described [160].

Free-living diazotrophs fix nitrogen without any cooperation with a plant host. FLNF bacteria are mostly heterotrophs, but autotrophs (such as *Cyanobacteriota*) are also present. From most plants’ perspective, the advantage of FLNF bacteria is that the benefits of this kind of fixation are accessible to all plants [128]. Free-living diazotrophs, including soil ones, face the most significant oxygen disadvantage, as ubiquitous O_2_ leads to irreversible inhibition of the nitrogenase complex [135]. As mentioned earlier in the context of *Cyanobacteriota*, oxygen avoidance strategies may consist of spatial isolation of nitrogenase from oxygen (as part of cell differentiation, or, e.g., as intracellular compartments), temporal isolation, growth strategy, production of biofilms as oxygen diffusion barriers, or increasing substrate utilisation (creating high-respiration, low-O_2_ soil microsites) [135,161].

There are both obligatory anaerobic, facultative aerobic, and obligatory aerobic free-living diazotrophs [162]. In general, FLNF can be found among various phyla, such as *Psuedomonadota* (*Alphaproteobacteria*, *Betaproteobacteria*, *Gammaproteobacteria*, *Deltaproteobacteria*, *Epsilonproteobacteria*), *Bacillota* (*Bacilli*, *Clostridia*), *Cyanobacteriota*, *Chlorobiota*, *Chloroflexota*, *Spirochaetota* [135,162].

Less studied than their counterparts, free-living diazotrophs play a significant role in global terrestrial BNF, contributing approx. one-third of it [138,140]. In environments with sparse vegetation, such as polar regions, free-living diazotrophs participate in nitrogen fixation by integrating into cryptogamic covers [138]. Cryptogamic communities consist of mosses, lichens, fungi, cyanobacteria, and algae. Unlike symbiotic diazotrophs, free-living ones are not confined to environments where host plants can thrive, making them potentially more important for BNF in cold and arid regions [138,140].

In addition to diazotrophic bacteria, nitrifying, denitrifying, anammox, and commamox bacteria also interact with plants and form part of their microbiome. Plants themselves can control the metabolic activity of these organisms through the phenomena of biological nitrification inhibition (BNI) and biological denitrification inhibition (BDI) [163,164,165].

Genetic markers related to nitrogen fixation with potential applications in high-throughput sequencing of marker genes, gene expression studies, metabolic potential screening, community screening, biomonitoring, (meta)genomic and (meta)transcriptomic data mining are presented in Table 4. The table also includes the possibly universal primer sets available for some marker genes based on analysis of the literature data.

### 5.2. Phosphorus

Phosphorus is the second most vital nutrient for plant growth after nitrogen, playing a critical role in various essential metabolic processes within the plant [181,182]. These processes include but are not limited to photosynthesis, cell development, cell division, respiration, energy transfer, and the biosynthesis of macromolecules [182,183]. Only a tiny fraction of total soil phosphorus (approximately 0.1%) is accessible for plant uptake [183,184]. This limited availability is attributed to various factors such as precipitation with soil cations, immobilisation, adsorption, and conversion to organic forms [183]. Plant roots can absorb phosphorus as orthophosphates, specifically in the forms of H_2_PO_4_^−^ or HPO_4_^2−^. However, these concentrations in the soil exist within the micromolar range [185].

Addressing the challenge of soil phosphorus deficiency has involved the application of phosphorus synthetic fertilisers [181]. However, a significant portion of these precipitate in soil. As soil contains various heavy metals, this leads to their accumulation. It also adversely affects soil fertility, the health of humans and other animals, eutrophication, and an expanded carbon footprint [183].

Phosphorus in soils occurs both in inorganic and organic forms. There is considerable ambiguity in the source data on the organic phosphorus content relative to the total pool: some estimates are over 20–30%, others 30–50%, and the content can vary from 5% to 95% [183,184]. Soil organic P is classified into three groups, depending on the phosphorus bonds: (i) phosphate esters; (ii) phosphonates; and (iii) phosphoric acid anhydrides [183,186]. Phosphate esters can be divided into two groups: phosphate monoesters (mainly inositol phosphates) and phosphate diesters (such as nucleic acids, phospholipids, and teichoic acids) [186]. Phosphonates differ from other organic forms due to the presence of a carbon-phosphorus bond (C–P) instead of a C–O–P bond. Examples of phosphoric acid anhydrides (organic polyphosphates) are adenosine diphosphate (ADP) and adenosine triphosphate (ATP) [186]. Phosphate monoesters are the predominant form of organic phosphorus in soils under aerobic conditions; phosphate diesters are less abundant than monoesters [186]. Inositol hexaphosphate (phytate) typically constitutes 30–50% of total organic phosphorus in soil [102]. It is the principal storage form of phosphorus in plants; yet, they are indigestible by humans and nonruminant animals and unavailable for plant roots (due to very low levels of phytases) [6]. Phosphonates tend to accumulate in wet, cold, or acidic soils with few phosphonate enzyme conditions; organic polyphosphates are present in trace amounts [186]. The inorganic phosphorus in the soil consists of phosphates bound to minerals such as calcium phosphate, aluminium phosphate, and iron phosphate [182,183]; moreover, inorganic polyphosphates, though less studied and underexplored, may also be present [187].

Phosphorus-solubilising bacteria (PSBs) can solubilise inorganic and/or mineralise organic insoluble phosphate compounds [183,185]. Inorganic phosphorus can be solubilised via the production of organic acids, inorganic acids, proton extrusion, EPS production, and, in the case of inorganic polyphosphates, enzymatically [183,187,188,189]. Organic acid production seems to be the primary mechanism. These organic acids solubilise inorganic P, as they (i) lower the pH, (ii) enhance chelation of the P-bound cations, (iii) challenge P for adsorption sites, and/or (iv) form soluble complexes with metal ions associated with insoluble P (Ca, Al, Fe) and thereby release the phosphorus [184]. One of the most common agents are gluconic acid (GA) and 2-ketogluconic acid [183,184,188,190]. The secretion of gluconic acid is mediated by glucose dehydrogenase, which is a quinoprotein involved in the direct oxidation pathway of glucose and encoded by the *gcd* gene [183,191]. The enzyme utilises pyrroloquinoline quinone (PQQ), a redox-active molecule produced by PQQ synthase encoded by the *pqq*ABCDEF gene cluster, as a cofactor [192,193]. The most extensively studied enzyme in the pathway is pyrroloquinoline quinone synthase C (PqqC), responsible for catalysing the last step of PQQ biosynthesis [189]. These genes are crucial in dehydrogenase activity and mineral phosphate solubilisation in microorganisms [183,191,193,194]. In different organisms, various other genes involved in GA production have been described, such as *mps* (mineral phosphate-solubilizing, e.g., in *Pantoea agglomerans*) and *gab*Y (e.g., in *Burkholderia cepacia*) [183,184,185,193,195].

Chemolithoautotrophic PSBs have been reported to produce hydrochloric acid, sulfuric acid, nitric acid, and carbonic acid, which can solubilise phosphate, but their efficiency is notably lower compared to organic acids [182,183]. PSBs can also lower the pH and, thus, solubilise phosphates by proton extrusion without producing acids [182]. NH_4_^+^ found in soil is assimilated by PSBs to synthesise amino acids. Within the microbial cell, NH_4_^+^ is transformed into NH_3_, and the excess protons (H^+^) are released into the cytoplasm. This process leads to acidification of the environment surrounding the microbial cell [183]. Inorganic phosphorus can be solubilised by exopolysaccharides (EPS sugars), as they form complexes with metal ions [188].

Finally, inorganic polyphosphates can be solubilised enzymatically. This includes inorganic pyrophosphatase (encoded by *ppa* gene) hydrolysing poly-P compounds and exopolyphosphatase (encoded by *ppx*), releasing inorganic P from poly-P chains [189].

Organic phosphates are solubilised in three ways by (i) non-specific acid phosphatases (NSAPs), (ii) phytases, and (iii) phosphonatases and C–P lyases [181,182,183]. Depending on their optimum pH, NSAPs can be divided into acidic and alkaline phosphatases. Acidic phosphatases (APs) are encoded in bacteria by *aph*A and *pho*N genes, alkaline (ALPs), by *pho*A, *pho*D, and *pho*X [189,196]. *pho*D is the most commonly found gene encoding phosphatase in soil, which explains why it is the primary research marker [189].

*pho*A, *pho*D, *pho*N, and *pho*X, among others, are members of the Pho regulon. This P starvation machinery generally consists of extracellular enzymes capable of acquiring P from organic phosphates, P-specific transporters, and enzymes involved in P storage and saving [197]. The Pho regulon is controlled by a two-component regulatory system consisting of an inner-membrane histidine kinase sensor protein and a cytoplasmic transcriptional response regulator [197]. These proteins are identified by various names in different bacteria, such as PhoR–PhoB in *Escherichia coli* and PhoR–PhoP in *Bacillus subtilis* [197].

Phytases (encoded by *app*A and *phy* genes) specifically catalyse P removal from phytate [183,188]. These enzymes can be categorised based on (i) their optimum pH (acid and alkaline phytases), (ii) their catalytic mechanisms, including histidine acid phytases, β-propeller phytases, protein tyrosine phosphatase-like phytases (also known as cysteine phytases), and purple acid phytases, and (iii) the carbon atom, where phosphate hydrolysis begins in the myo-inositol ring, with 3-, 4-, and 5-phytases being the most recognised [189,198]. Phosphonatases and C–P lyases catalyse the cleavage of the C–P bond of organophosphates [182,183,184]. The C–P lyase activity is attributed to the gene products of the phn operon (consisting of 14 genes) [189]. Among these, PhnG, PhnH, PhnI, PhnJ, PhnK, PhnL, and PhnM proteins constitute the essential catalytic set for C–P lyase activity [189]. This enzymatic cleavage of relatively stable C–P bonds can also be performed by phosphonoacetaldehyde hydrolases (encoded by the *phn*X gene) [173,199].

Table 4 presents genetic markers related to inorganic and organic phosphorus solubilisation/mineralisation with potential amplicon HTS applications, gene expression studies, screening of metabolic capabilities, biomonitoring, and (meta)genomic and (meta)transcriptomic data mining. The table also includes the possibly universal primer sets.

Li et al. have summarised that, as of 2021, 2286 bacterial strains have been reported to be PSBs [200]. Twenty-five bacterial genera were identified as rich in PSBs, each having more than ten strains. *Bacillus*, *Pseudomonas*, *Enterobacter*, and *Burkholderia* had over 100 identified PSB strains, making them significant PSB genera [200]. The vast majority of PSBs could solubilise inorganic phosphorus, about ten times less mineralised organic phosphorus. Only a few dozen strains could transform both inorganic and organic phosphorus, most of which were *Paenibacillus*, *Bacillus*, *Pseudomonas*, *Lactococcus*, *Enterobacter*, and *Alcaligenes* [200]. Those six were also considered significant PSB genera (three of them overlapped with the >100 species genera) [200]. Altogether, seven bacterial strains were deemed essential, all belonging to *Bacillota* or *Pseudomonadota*.

On the other hand, the genetic potential to solubilise inorganic and/or mineralise organic phosphorus has been described for a wide range of bacteria, including *Pseudomonadota*, *Actinomycetota*, *Bacillota*, *Bacteroidota*, *Cyanobacteriota*, and *Fusobacteriota* [200]. Li et al. have analysed all available (as of 2019) complete bacterial and archaeal genomes looking for GDH (glucose dehydrogenases), APs, ALPs, and phytases [200]. Among 12986 genomes, 1524, 4367, 6377, and 2401 were found to have GDH-, AP-, ALP-, and phytase-positive genotypes, respectively. Based on the genetic potential, the authors selected the six most promising genera: *Klebsiella*; *Xanthomonas*; *Enterobacter*; *Serratia*; *Acinetobacter;* and *Pseudomonas*. Interestingly, one can notice that in genetic predictions, bacteria with the genetic potential for solubilising organic phosphates outnumbered those of inorganic phosphates, i.e., opposite to the analyses of strains described in the literature as PSBs [200]. It seems that most PSBs are yet to be characterised.

### 5.3. Potassium

Potassium, along with nitrogen and phosphorus, is a pivotal essential nutrient for plants, constituting the third element within the traditional NPK chemical fertilisers [201]. K emerges as the predominant cation within plant tissues, primarily as a free ion. K^+^ plays an essential role in cell metabolism, growth, and development. The long-distance transport of K^+^ is crucial for balancing inorganic and organic anions, serving as the dominant cation in the xylem and phloem [202]. Within plant cells, K^+^ facilitates the transport of organic anions and metabolites, maintains transmembrane voltage gradients for cytoplasmic pH homeostasis, supports protein synthesis, aids in photosynthesis, regulates osmotic balance, and contributes to cell extension [202].

Four primary K pools have been identified within the soil: (i) soil solution K (plant-available); (ii) exchangeable K (readily available), (iii) slowly exchangeable or fixed K (slowly available), (iv) structural or lattice K (unavailable) [202]. In general, the soil contains abundant K reserves, yet only a tiny fraction (1–2%) is available or readily available for direct absorption by plants [203]. The vast majority, comprising 90% to 98% of soil potassium, exists in various forms of silicate minerals. These minerals include orthoclase, feldspar, biotite, mica, vermiculite, muscovite, smectite, illite, and others [203].

KSBs—potassium-solubilizing bacteria—act mainly via the production of organic acids, such as citric, tartaric, succinic, 2-ketogluconic, and oxalic acids [203,204]. They dissolve potassium, silicon, and aluminium from insoluble K-bearing minerals. Organic acids directly dissolve potassium from the rock or chelate silicon, iron, and aluminium ions, bringing potassium into the solution [203,204]. It seems that EPS can also dissolve K-bearing minerals. Polysaccharides can absorb organic acids bound to the surface of silicate minerals, leading to a high concentration of acids surrounding the mineral [204]. Researchers have proposed that EPS plays a role in absorbing SiO_2_, thereby influencing the equilibrium between the mineral and fluid phases and, as a consequence, promoting SiO_2_ and K solubilisation [204].

KSBs have been described within the following phyla: *Pseudomonadota* (in *Rhizobiaceae*, *Burkholderiaceae*, *Alcaligenaceae*, *Enterobacteriaceae*, *Pseudomonadaceae*, *Lysobacteraceae* families); *Bacteroidota* (in *Cytophagaceae*, *Flavobacteriaceae*); *Bacillota* (in *Bacillaceae*, *Paenibacillaceae*); *Actinomycetota* (*Micrococcaceae*, *Microbacteriaceae*) [201]. As of 2024, there is no literature data on KSB-specific marker genes and, thus, no published genetic predictions.

## 6. Siderophores as a Shared Phenomenon

### 6.1. Molecular Characteristics

Iron plays a key role in various processes both within plants and bacteria. Like most organisms, plants and bacteria need iron as a key cofactor in multiple enzymes [205,206]. Although iron is abundant in the Earth’s crust, it is mostly not bioavailable, as it exists mainly in an oxidised Fe^3+^ state, characterised by its insolubility in neutral and alkalic pH levels [205,207]. A prevalent bacterial method for accumulating iron is the production of siderophores. Siderophores are low-molecular-weight compounds possessing a high affinity and selectivity for Fe^3+^ [205]. There are hundreds of different siderophores described; it seems that almost all known bacterial species produce siderophores; different organisms can produce the same siderophores, and single organisms can produce multiple different siderophores; its biosynthesis is typically regulated by the iron levels in the environment [185,205,207,208].

All siderophores exhibit a stronger affinity for Fe^3+^ compared to Fe^2+^. As noticed by Hider and Kong [205], there is sound logic in this selectivity, as it is challenging to create ligands that specifically target Fe^2+^ (over biologically significant dipositive cations such as Zn^2+^, Cu^2+^, Ni^2+^, Mn^2+^), but it is much less challenging in the case of tripositive cations, as there are not many biologically important cations of this type, so ligands selective for tripositive metals will, at the exact moment in biological matrices, be selective for iron [205]. Nevertheless, many siderophores act as zincophores and form complexes involved in transporting manganese in the form of Mn^3+^, e.g., in manganese-oxidising bacteria [209,210]. Furthermore, siderophores are also crucial in the context of nitrogen fixation; it has been described that some bacteria produce siderophores under Mo- and V-limiting conditions to acquire the cofactors necessary for the nitrogenase complex [209].

**Figure 4:**
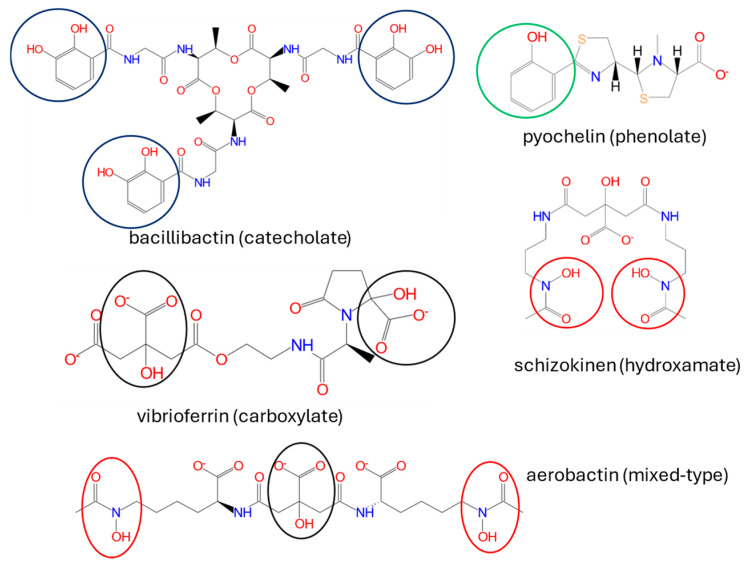
Examples of siderophores belonging to different groups. Inside the circles, the functional groups based on which the siderophores are classified are shown (in the case of mixed-typed siderophores, these are example groups). Graphics from the MetaCyc database [213] were used.

Siderophores are grouped based on their typical ligands; however, they are grouped in different manners. Classifications always include three groups: (i) catecholate; (ii) hydroxamate; and iii) (α-hydroxy)carboxylate ones. Some authors distinguish three groups only (see [211]), others—four. According to Kramer et al. [207], siderophores should be divided into (i) catecholate, (ii) phenolate, (iii) hydroxamate, and (iv) (α-hydroxy)carboxylate ones; however, according to Timofeeva et al. [206], catecholate and phenolate should be treated as one group, and a fourth group should include mixed-type siderophores. Based on this, some authors distinguish not four but five main groups (Figure 4) [212]. Moreover, it is worth noticing that other ligands, such as hydroxyphenyloxazolone, α-aminocarboxylate and α-hydroxyimidazole, are also present in siderophores [205,208].

Synthesis of bacterial siderophores can take place in three ways: by non-ribosomal peptide synthases (NRPSs); by polyketide synthases (PKSs); and by NRPS-independent siderophore synthases (NISs) [206,214]. NRPS are large multi-domain and multi-enzyme complexes, each unit responsible for the attachment of one amino acid (aa) to a developing peptide chain, including non-proteinogenic aa and hydroxy acids. Typically, NRPSs are composed of adenylation (A), condensation (C), peptidyl carrier protein (PCP), and thioesterase (TE) domains, but other domains, such as epimerisation (E), oxidation (Ox), methylation (Mt), and cyclisation (Cy) may also occur [185,215,216]; biosynthesis is a three-step process. PKSs are made of acyltransferase (AT), acyl carrier protein (ACP), ketosynthase (K), and thioesterase (TE) domains. As for NRPSs, modifications, such as ketoreductase, dehydratase, methyltransferase, and oxidation domains, may occur [185]. The abovementioned modular structure enables biosynthetic gene clusters (BGCs) detection and, thus, prediction of NRPS and PKS-dependent secondary metabolites, e.g., by using software such as the antiSMASH [180]. Nevertheless, as noted by McRose et al. [208], flexibility in the incorporation of siderophore precursors and the release of small fragments or precursors make it challenging to predict the complete range of siderophore structures produced by an organism (without direct analysis).

Siderophores enable iron acquisition through specific uptake systems. The release of these molecules is facilitated by energy-dependent mechanisms and is mediated by efflux pumps belonging to different superfamilies (depending on the transported siderophore type) [207]. When it comes to the uptake of siderophores in Gram-negative bacteria, various siderophore-mediated iron uptake pathways have been identified, comprising an outer membrane receptor, a periplasmic binding protein, and a complex consisting of one or two cytoplasmic membrane proteins associated with an ATP-binding cassette (ABC), forming a complete carrier system [185]. The transport of siderophores across the outer cell membrane is mediated by a complex of three transmembrane proteins: TonB; ExbD; and ExbB [185].

Notably, most bacteria have separate systems for different siderophores and systems for secretion of siderophores produced by competing microorganisms [185,207]. As Gram-positive bacteria lack the outer membrane and the corresponding receptors, Fe^3+^-siderophore complexes are captured by periplasmic siderophore-binding proteins bound to the plasma membrane. Subsequently, these complexes are transported into the cytoplasm through the ABC transport system, as in Gram-negative bacteria [185]. Then, inside cells, Fe^3+^ is released from the complex, becoming available for cellular processes. Two main mechanisms have been described: the first involves reducing Fe^3+^ to Fe^2+^, followed by spontaneous release or competitive binding; the second uses specialised hydrolases, destabilising the complex [185]. After the bacteria have accumulated a sufficient amount of iron, the ferric uptake regulator (Fur) acts either directly as a transcriptional repressor of Fe-uptake genes (and uses Fe^2+^ as a co-repressor) or indirectly as a protein regulatory activator [205,207,217,218]. Fur protein is conserved in multiple bacteria, both Gram-negative and Gram-positive ones [217,218].

Bacteria can produce several or many types of siderophores. This phenomenon is known as multiple siderophore production (MSP). It is based on three basic mechanisms: (i) utilisation of biosynthetically distinct gene clusters; (ii) release of precursors and partial products; and (iii) incorporation of variable precursors during biosynthesis [208]. In the first case, organisms encode multiple biosynthetic gene clusters responsible for producing different types of siderophores. In the second situation, an organism encodes genes for synthesising a typically large siderophore, but smaller fragments of this compound accumulate during growth. Lastly, alterations in intracellular metabolite pools direct the incorporation of diverse siderophore precursors into the final product [208]. It is worth mentioning that abiotic photochemical reactions can also generate multiple siderophores; however, this is beyond the scope of this review paper. The functions and eco-evolutionary context of MSP will be discussed in the next section.

As mentioned, siderophore-producing bacteria are ubiquitous [207]. Siderophores have been well described in at least four phyla (*Actinomycetota*, *Bacillota*, *Cyanobacteriota*, *Pseudomonadota*), in at least five classes: *Actinomycetes* (e.g., *Nocardia*, *Rhodococcus*, *Streptomyces*); *Bacilli* (e.g., *Bacillus*, *Paenibacillus*); *Cyanophyceae* (e.g., *Agmenellum*, *Anabaena*, *Anacystis*, *Gloeocapsa*, *Microcystis*, *Oscillatoria*, *Phormidium*, *Spirulina*, *Synechococcus*); *Alphaproteobacteria* (e.g., *Azospirillum*, *Methylobacterium*); and *Gammaproteobacteria* (e.g., *Azotobacter*, *Dickeya*, *Enterobacter*, *Klebsiella*, *Kosakonia*, *Pantoea*, *Pseudomonas*, *Serratia*) [206,219].

In bacteria–plant host interactions, the most obvious role is to increase iron bioavailability [185,207]. Nevertheless, despite several competing hypotheses, no exact molecular mechanism has been experimentally confirmed [185]. Siderophores are also crucial in abiotic stress (they protect against oxidative stress) and biotic stress, as siderophore-mediated competition may lead to plant-defending bacteria locking iron away from pathogens and, thus, preventing their growth [207]. In addition, there are antibiotics, known as sideromycins, distributed by binding to siderophores [207].

### 6.2. Siderophores Revisited—Microbial Community Questions

Like any biological phenomenon, siderophore production and iron uptake can be considered in two categories: the proximate (how?), concerning mechanistic aspects, and the ultimate (why?), concerning evolutionary and adaptive aspects [207]. The biosynthesis of siderophores has already been discussed, but several ‘why?’ questions arise. Firstly, why do bacteria develop diffusible siderophores despite the losses incurred through diffusion? Why do they not depend solely on membrane-bound iron uptake systems, which are present in many taxa? And secondly, why do bacteria produce so many different types of siderophores?

The first issue needs to be considered in several contexts: from the perspective of a single cell, from the perspective of social interactions, and eco-evolutionary dynamics.

From a single-cell perspective, following Kramer et al. [207], we can consider four basic scenarios: for motile and surface-attached cells and different environmental structures. It seems that the membrane-bound systems are more efficient for motile cells and homogeneous iron distribution, and the siderophores do not give an advantage. If bacteria under the same conditions are surface-attached, siderophores can access iron beyond the local pool, but there are still many losses associated with diffusion. However, if the environmental structure is different, when the iron resources are clumped, and the bacteria are not directly next to the iron source, the motile bacteria-producing siderophores need not have surface-dependent contact with iron. Finally, if the iron resource is clumped and the cell is surface-attached, then siderophores may be the only method of obtaining iron at all [207].

Of course, bacteria do not exist as single cells but as groups. Thus, cells depend not only on their siderophores but also on the siderophores of companions (most often clonemates) [207]. Siderophore sharing provides a tool for compensating for diffusion losses, both in motile cells and surface-attached cells (e.g., in biofilms).

However, as siderophores are extracellular products, they are public and leaky, i.e., their release may create favourable conditions for other strains and species [220,221]. Thus, any siderophore-producing bacterial population can be exploited by cheaters who avoid production costs while still reaping the benefits others provide. This leads to the so-called Black Queen (BQ) hypothesis. According to this, loss of function mutation leads to the emergence of non-producing ecotypes, i.e., the beneficiaries (cheaters). Cheaters do not produce siderophores but still have matching receptors for uptake and, thus, exploit the common pool without any contribution [207]. BQ taxa, i.e., the passive helpers, were too slow to lose functions and hence were forced to carry out costly siderophores biosynthesis for the mutual benefit [222].

**Figure 5:**
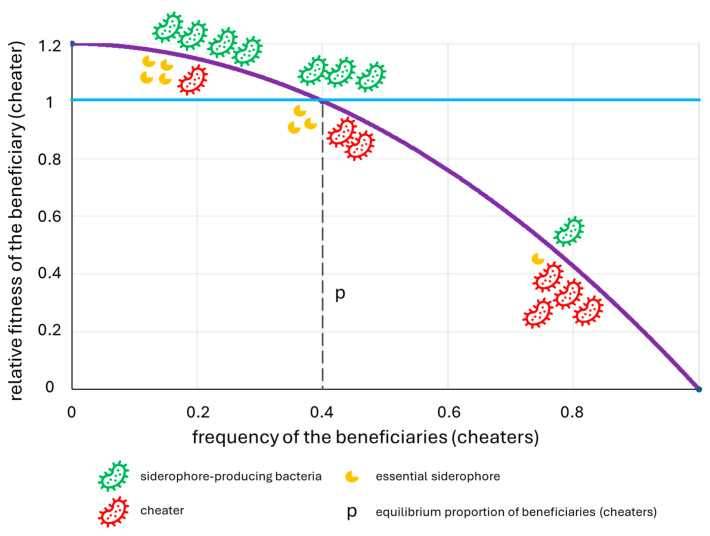
The depiction of negative frequency-dependent selection based on schemes of Morris et al. and Kramer et al. [207,221].

Negative frequency-dependent selection is typically observed in a system characteristic of the BQ hypothesis (Figure 5). As the frequency of beneficiaries (cheaters) increases, their relative fitness decreases due to the lack of leaked products [207,220,221]. This regulation makes the long-term coexistence of passive helpers and cheaters possible [220,221].

Sometimes, the BQ arrangement can lead to the so-called Tragedy of the Commons (ToC). When modelled for two bacterial species, ToC represents a total collapse of the system due to the overutilisation of products by cheaters [207,221,222]. In the practice of multi-species interactions, ToC is understood as a situation in the community where the excessive presence of taxa that do not contribute to the production of public goods hinders collective growth [207,221,222]. Finn et al. [222] performed simulations for bacteria in rhizosphere and bulk soil. In the case of bulk soil, cooperativity and oligotrophy were promoted, while accumulation of loss of function mutants led to ToC risk. In the case of rhizosphere host-associated communities, copiotrophy and cheating were rewarded [222].

In addition to cooperation and cheating, competition is also typical. Siderophores can serve as competitive agents against other bacteria; this underlies the MSP phenomenon [207,208]. According to the classic Red Queen (RQ) hypothesis, it leads to an ‘arms race’ [221].

Production of different types of siderophores results in the locking away of non-producers (that lack the matching uptake receptors), avoiding siderophore theft, and rivalry with other siderophore producers [207,208]. The simultaneous production of siderophores with varying iron affinities could improve Fe dissolution via ligand exchange, and the coexistence of hydrophilic and hydrophobic siderophores could function in a so-called bucket brigade mechanism, where more hydrophilic siderophores scavenge Fe and transfer it to more hydrophobic ones [208]. As noted by McRose et al., multiple siderophores exist because they serve a function: they work synergistically or contingently to provide Fe to the bacterial cell and/or have important non-Fe-related roles [208]. Cheaters can also obtain new traits, for example, by acquiring genes responsible for receptors that bind foreign siderophores not produced by the particular species [207,220].

It is also essential to look at the significance of different MSP mechanisms. Encoding multiple biosynthetic gene clusters enables the transcriptional regulation of siderophores in response to, e.g., Fe concentration [208]. Yet, the release of siderophore fragments could have been selected in an evolutionary timeline. The third mechanism, connected to precursor-directed biosynthesis, directly links siderophore production to environmental and metabolic conditions, circumventing both the heightened specificity and limitations of transcriptional regulation [208].

The evolutionary arms race may lead, inter alia, to the non-transitive community dynamics, the non-hierarchical circular competitive relationships among species, where each species is both superior and inferior to different members of the community, with no single overall winner prevailing in the population [207]. This can be explained by the example of Kramer et al. [207]: let us imagine a cheater outcompetes siderophore producer 1 (due to the siderophore theft). Yet, siderophore producer 1 outcompetes siderophore producer 2 (due to the efficacy of siderophores). In contrast, siderophore producer 2 outcompetes the cheater because it produces a siderophore that the non-producer lacks the corresponding receptor. This results in a scenario where there is no overall winner [207].

Is there a state of equilibrium between Red Queen and Black Queen, between the arms race, in which the loser loses access to resources, and the race to the bottom, in which the loser is forced to maintain a leaky function and act as a passive helper? In the context of genome complexity, RQ and BQ strategies can be viewed as moving in opposite directions: Red Queen races tend to enhance genome size and complexity, while Black Queen races result in smaller, simpler genomes. Some have proposed that loss of function is the primary mode of evolution, with occasional bursts of rapid complexity and genome size expansion (triggered by short RQ competitions) interrupting prolonged phases of continuous simplification through BQ functions and other mechanisms [221]. However, it should be remembered that different microenvironments may promote different evolutionary strategies, as demonstrated by Finn et al. [222].

## 7. Concluding Remarks

Times of rapid advances in omic techniques and biodiversity studies, along with the critical need for environmentally friendly solutions in agriculture and combating invasive species, require several solutions and clarifications, which this article sought to address.

We indicated a strong need to standardise the terminology of omic approaches. A clear distinction between metagenomics and metataxonomics, a summary of often mixed terms such as microbiome or microbiota, an analysis of the validity of the use of the terms multi-omics and pan-omics, or finally, a discussion of the concept of holobiont and, thus, the idea of holo-omics is crucial for an orderly scientific debate. Naturally, they are probably clear to specialists in their niche, but omics methods are becoming widespread across disciplines, and the terminological chaos does not support their widespread use.

The diversity of the rhizosphere s.l. is by far the best studied, while the aboveground parts, especially seeds and flowers, are less well understood. In the case of seeds, meta-analyses/datasets have come out in the very last few years. In the case of the anthosphere microbiome, we discovered a lack of datasets on the structure and diversity of this part of the plant holobiont, even at the basic level of taxonomic analyses. We created the first data compilation in a decade concerning flower microbiome diversity studied by culture-independent methods and the first in general regarding depth of analysis.

Throughout the work, we wanted to emphasise that it is not the binary plant-bacteria interactions but the totality of interactions within the plant microbiome that influences the functioning of the plant host. Furthermore, many of the members of this microbiota are involved in nitrogen fixation, as well as phosphorus and potassium cycling. Nitrogen-fixing organisms are more diverse than previously thought, and new types of interactions and molecular details are still being described, and many of the phosphorus-transforming organisms remain to be discovered. Above all, however, we wanted to highlight the remarkable understudying of potassium solubilisation and the absence of molecular markers, which is particularly surprising given that NPK fertilisers are widely used.

Moreover, genetic markers concerning nutrient acquisition were also compiled. This can be used in (meta)genome and (meta)transcriptome mining, in HTS of selected marker genes, gene phylogenies, gene expression studies, screening of strains for metabolic abilities, biomonitoring for the presence of genes involved in element transformation processes, and quantification of those organisms.

We also described how siderophore production is a two-faced phenomenon of molecular and cellular biology and microbial ecology. Other processes are still waiting to be investigated together from these perspectives, and relying on only one of them is impossible in the age of microbiological advances.

As the authors, we would like to highlight the future potential of using the microbiome of compartments other than underground parts, especially seeds and flowers. From a broader perspective, we would like to point out that the research on plant–microbiome–environment interactions is one of the key scientific frontiers in the context of contemporary anthropogenic change and can make essential contributions to frameworks and concepts such as Planetary Boundaries, One Health, One Earth, Biosecurity, Sustainable Development Goals, Regenerative Agriculture, and Nature-based Solutions. We hope this work can inspire in times of unprecedented challenges and growing efforts towards designing and applying whole microbial communities.

## Figures and Tables

**Table 2:** The stoichiometry of biological nitrogen fixation by the three nitrogenase isoforms [134].

Nitrogenase Containing:	Reaction Stoichiometry:
MoFe protein	N_2_ + 8 H^+^ + 16 MgATP + 8 e^−^ → 2 NH_3_ + H_2_ + 16 MgADP + 16 P_i_
VFe protein	N_2_ + 12 H^+^ + 24 MgATP + 12 e^−^ → 2 NH_3_ + 3 H_2_ + 24 MgADP + 24 P_i_
FeFe protein	N_2_ + 21 H^+^ + 40 MgATP + 20 e^−^ → 2 NH_3_ + 7 H_2_ + 40 MgADP + 40 P_i_

**Table 3:** All types of rhizobia so far described, according to Wang [144]. All names are according to LPSN [39].

Phyla	Classes	Order	Family	Genus
*Pseudomonadota*	*Alphaproteobacteria*	*Hyphomicrobiales*	*Rhizobiaceae*	*Agrobacterium*
				*Allorhizobium*
				*Ensifer*
				*Neorhizobium*
				*Pararhizobium*
				*Rhizobium*
				*Shinella*
			*Phyllobacteriaceae*	*Aminobacter*
				*Phyllobacterium*
				*Mesorhizobium*
			*Nitrobacteraceae*	*Bradyrhizobium*
			*Methylobacteriaceae*	*Microvirga*
				*Methylobacterium*
			*Brucellaceae*	*Brucella*
			*Devosiaceae*	*Devosia*
			*Xanthobacteraceae*	*Azorhizobium*
	*Betaproteobacteria*	*Burkholderiales*	*Burkholderiaceae*	*Cupriavidus*
				*Paraburkholderia*
				*Trinickia*
			*Oxalobacteraceae*	*Herbaspirillum*

**Table 4:** Compilation of genetic markers for nitrogen fixation processes and solubilisation or mineralisation of phosphorus compounds. Sequences of potentially universal marker-specific primers are also included. In addition, information on potassium and iron is included. N.A.—not available; N.I. – not included.

Process	Gene	Protein [E.C. Number] [KO Number]	Description	Universal Primers	
				Primer Pairs (Sequences)	1st Publication
nitrogen fixation	*nif*H	dinitrogenase reductase (Fe protein) [K02588]	MoFe nitrogenase enzyme complex	Ueda19F/R6 (5′-GCIWTYTAYGGIAARGGIGG-3′/ 5′-GCCATCATYTCICCIGA-3′), IGK3/DVV (5′-GCIWTHTAYGGIAARGGIGGIATHGGIAA-3′/5′-ATIGCRAAICCICCRCAIACIACRTC-3′) F2/R6 (5′-TGYGAYCCIAAIGCIGA-3′/ 5′-GCCATCATYTCICCIGA-3′); database and NifMAP bioinformatic pipeline available	Gaby and Buckley, 2014, Angel et al., 2018 [166,167]
	*nif*D	MoFe dinitrogenase subunit alpha [EC:1.18.6.1] [K02586]		FdB261/FdB260 (5′-TGGGGICCIRTIAARGAYATG-3′/ 5′-TCRTTIGCIATRTGRTGNCC-3′)	Stoltzfus et al., 1997 [168]
	*nif*K	MoFe dinitrogenase subunit beta [EC:1.18.6.1] [K02591]		sxnif_K1/sxnif_K2 (5′-CCTGGATGACCGAAGACGC-3′/ 5′-GGTGCCGCCTTCATACAT-3′)	Dai et al., 2014 [169]
	*vnf*H	dinitrogenase reductase (Fe protein) [K22899]	VFe nitrogenase enzyme complex	N.A.	
	*vnf*D	VFe dinitrogenase subunit alpha [EC:1.18.6.2] [K22896]		(*vnf*D, *anf*D): D1f/D5r 5′-CGGGATCCTCIGARCGIGGITGYGC-3′/ 5′-CGTCTAGAYTCYTTRTACCARTC-3′)	Loveless and Bishop, 1999 [170]
	*vnf*K	VFe dinitrogenase subunit beta [EC:1.18.6.2] [K22897]		N.A.	
	*vnf*G	VFe dinitrogenase subunit delta [EC:1.18.6.2] [K22898]		D6F/K3r (5′-CGTCTAGAYTCYTTRTACCARTC-3′/ 5′-GCAGTCGTACATCGGGTT-3′)	Loveless and Bishop, 1999, Betancourt et al., 2008 [170,171]
	*anf*H	dinitrogenase reductase (Fe protein)	FeFe nitrogenase enzyme complex	N.A.	
	*anf*D	FeFe dinitrogenase subunit alpha [EC:1.18.6.1]		(*vnf*D, *anf*D): D1f/D5r 5′-CGGGATCCTCIGARCGIGGITGYGC-3′/ 5′-CGTCTAGAYTCYTTRTACCARTC-3′)	Loveless and Bishop, 1999 [170]
	*anf*K	FeFe dinitrogenase subunit beta [EC:1.18.6.1]		N.A.	
	*anf*G	FeFe dinitrogenase subunit delta [EC:1.18.6.1] [K00531]		D7F/K2r (5′-GCTCTAGACGCSATCTAYTCGCCGA-3′/ 5′-CGGAATTCCGATGCAATCCTTGAT-3′)	Loveless and Bishop, 1999 [170]
	*nif*B/*nif*B I-IV	radical SAM enzyme catalyzing NifB-co ([8Fe-9S-C] cluster) formation [K02585]	essential for the synthesis of all nitrogenases	N.A.	
	*nif*EN	terminal assembly scaffold protein [K02587/K22903 + K02592]	essential for the synthesis of MoFe nitrogenases	no universal primers, but recently created for whole phylum (*Cyanobacteriota*): nifE-F/nifE-R (5′-CACCCAAGGCAAAATYAACG-3′/ 5′-CCCACATAACCWGCATAAGG-3′), nifN-F/nifN-R (5′-GTYAATCCYCTCAAGCAAAG-3′/ 5′-CCTAARCGGTCRTAAATGGG-3′)	Giannakopoulos et al., 2024 [172]
	*nif*EN/*vnf*EN		essential for the synthesis of VFe nitrogenases	N.A.	
organic phosphorus mineralisation	*app*A	phytase AppA [EC 3.1.3.26] [K01093]	inositol hexaphosphate (phytate) mineralisation	appA-FW/appA-RW (5′-AGAGGGTGGTGATCGTGATGMGICAYGGNRT-3′/ 5′-GCCTCGATGGGGTTGAAIADNGGRTC-3′)	Bergkemper et al., 2016 [173]
	*bpp*	β-propeller phytase [EC 3.1.3.8] [K01083]		BPP-F/BPP-R (5′-GACGCAGCCGAYGAYCCNGCNIT NTGG-3′/5′-CAGGSCGCANRTCIACRTTRTT-3′)	Huang et al., 2009 [174]
	*cphy*	ruminal cysteine phytase [EC 3.1.3.X]		Cphy-F/Cphy-R (5′-GTGGACCTRCGRMARGARWCICA-3′/5′-GTCCGACCATTGCCTGCYTCRCARTGRAMRT GIADCCA-3′)	Huang et al., 2011 [175]
	*pho*A	alkaline phosphatase A [EC 3.1.3.1] [K01077]	phosphate monoesters mineralisation	N.A.	
	*pho*D	alkaline phosphatase D [EC 3.1.3.1] [K01113]		ALPS-F730/ALPS-R1101 (5′-CAGTGGGACGACCACGAGG T-3′/5′-GAGGCCGATCGGCATGTCG-3′) phoD-FW/phoD-RW (5′-TGTTCCACCTGGGCGAYWMI ATHTAYG-3′/5′-CGTTCGCGACCTCGTGRTCRTCCCA-3′)	Sakurai et al., 2008, Bergkemper et al., 2016 [173,176]
	*pho*X	alkaline phosphatase X [EC 3.1.3.1]		PhoX2-F/PhoX2-R (5′-GARGAGAACWTCCACGGYTA-3′/5′-GATCTCGATGATRTGRCCRAAG-3′)	Sebastian and Ammerman, 2009 [177]
	*pho*N	acid phosphatase class A [EC 3.1.3.2] [K09474]		phoN-FW/phoN-RW (5′-GGAAGAACGGCTCCTACCC IWSNGGNCA-3′/5′-CACGTCGGACTGCCAGTGIDMIYY RCA-3′)	Bergkemper et al., 2016 [173]
	*aph*A	acid phosphatase class B [EC 3.1.3.2] [K03788]		N.A.	
	*olp*A	acid phosphatase class C [EC 3.1.3.2]		N.A.	
	*phn*G	alpha-D-ribose 1-methylphosphonate 5-triphosphate synthase subunit PhnG [EC:2.7.8.37] [K06166]	C–P lyase multienzyme complex; organic phosphonate mineralisation	N.A.	
	*phn*H	alpha-D-ribose 1-methylphosphonate 5-triphosphate synthase subunit PhnH [EC:2.7.8.37] [K06165]		N.A.	
	*phn*I	alpha-D-ribose 1-methylphosphonate 5-triphosphate synthase subunit PhnI [EC:2.7.8.37] [K06164]		N.A.	
	*phn*J	alpha-D-ribose 1-methylphosphonate 5-phosphate C–P lyase [EC:4.7.1.1] [K06163]		N.A.	
	*phn*K	putative phosphonate transport system ATP-binding protein [K05781]		PhnK-F/PhnK-R (5′-CATCGTCGGCGAATCCGG-3′/ 5′-TGCTGCATGCCGCCGGAAAA-3′)	Zheng et al., 2018 [178]
	*phn*L	alpha-D-ribose 1-methylphosphonate 5-triphosphate synthase subunit PhnL [EC:2.7.8.37] [K05780]		N.A.	
	*phn*M	alpha-D-ribose 1-methylphosphonate 5-triphosphate diphosphatase [EC:3.6.1.63] [K06162]		N.A.	
	*phn*X	phosphonoacetaldehyde hydrolase [EC:3.11.1.1] [K05306]	organic phosphonates mineralisation	phnX-FW/phnX-RW (5′-CGTGATCTTCGACtGGGC NGGNAC-3′/5′-GTGGTCCCACTTCCCCADICCCATNGG-3′)	Bergkemper et al., 2016 [173]
inorganic phosphorus solubilisation	*gcd*	quinoprotein glucose dehydrogenase [EC:1.1.5.2] [K00117]	production of gluconic acid, unspecific yet strongly related	gcd-FW/gcd-RW (5′-CGGCGTCATCCGGGSITIYRAYRT-3′/5′-GGGCATGTCCATGTCCCAIADRTCRTG-3′, gcd-F/gcd-R (5′-ATCGCGTTCGGGCCGGACG-3′/ 5′-ATSAGRTTSAGCTCGTCCCA-3′)	Bergkemper et al., 2016, Zheng et al., 2018 [173,178]
	*pqq*C	pyrroloquinoline-quinone synthase [EC:1.3.3.11] [K06137]	production of gluconic acid, unspecific yet strongly related (indirectly)	PqqC-F/PqqC-R (5′-AACCGCTTCTACTACCAG-3′/ 5′-GCGAACAGCTCGGTCAG-3′)	Zheng et al., 2017 [179]
	*ppa*	inorganic pyrophosphatase [EC:3.6.1.1] [K01507]	inorganic polyphosphate solubilisation	N.A.	
	*ppx*	exopolyphosphatase [EC:3.6.1.11] [K01514]		Ppx-F/Ppx-R (5′-TGCATCTGGCGGACGGCCT-3′/ 5′-AGATCCGCCGCCAATATCA-3′)	Zheng et al., 2018 [178]
potentially inorganic phosphorus solubilisation and potassium solubilisation	genes related to the production of various organic acids	various enzymes	unspecific but related to both phosphorus and potassium solubilisation	N.I.	
metallophore/siderophore and pulcherriminic acid production	NRPS-, PKS-, and NIS-related genes	non-ribosomal peptide synthases (NRPSs), polyketide synthases (PKSs), NRPS-independent synthases		possible to detect and potentially design with software such as AntiSMASH	Blin et al., 2023 [180]
